# CoVox: A dataset of contrasting vocalizations

**DOI:** 10.3758/s13428-025-02664-9

**Published:** 2025-04-11

**Authors:** Camila Bruder, Pauline Larrouy-Maestri

**Affiliations:** 1 Max Planck Institute for Empirical Aesthetics, Grüneburgweg 14, 60322 Frankfurt Am Main, Germany; 2 Center for Language, Music, and Emotion (CLaME), New York, NY USA

**Keywords:** Voice, Singing, Speech, Corpus, Acoustics

## Abstract

The human voice is remarkably versatile and can vary greatly in sound depending on how it is used. An increasing number of studies have addressed the differences and similarities between the singing and the speaking voice. However, finding adequate stimuli material that is at the same time controlled and ecologically valid is challenging, and most datasets lack variability in terms of vocal styles performed by the same voice. Here, we describe a curated stimulus set of vocalizations where 22 female singers performed the same melody excerpts in three contrasting singing styles (as a lullaby, as a pop song, and as an opera aria) and spoke the text aloud in two speaking styles (as if speaking to an adult or to an infant). All productions were made with the songs’ original lyrics, in Brazilian Portuguese, and with a/lu/sound. This ecologically valid dataset of 1320 vocalizations was validated through a forced-choice lab experiment (*N* = 25 for each stimulus) where lay listeners could recognize the intended vocalization style with high accuracy (proportion of correct recognition superior to 69% for all styles). We also provide acoustic characterization of the stimuli, depicting clear and contrasting acoustic profiles depending on the style of vocalization. All recordings are made freely available under a Creative Commons license and can be downloaded at https://osf.io/cgexn/.

**Supplementary Information:**

The online version contains supplementary material available at 10.3758/s13428-025-02664-9.

The human voice can produce a wide range of sounds that serve multiple functions. Numerous studies have examined the similarities and differences between speech and music/song (e.g., Bradley, 2018; Livingstone et al., 2013; Merrill & Larrouy-Maestri, 2017), across cultures (Albouy et al., 2024; Anikin et al., 2023; Kachlicka et al., 2023; Ozaki et al., 2024) and in terms of neural processing (e.g., Albouy et al., 2020; Harris et al., 2023; Lévêque & Schön, 2015; Norman-Haignere et al., 2022; Tierney et al., 2013; Whitehead & Armony, 2018). These studies have collectively identified distinct acoustic patterns for speech and song. For example, songs typically exhibit higher pitch height and pitch stability and a slower temporal rate compared to speech (Ozaki et al., 2024), as well as higher regularity, which allows for the perception of a beat and more repetition than speech (Margulis, 2013; Yu et al., 2023). Also, recent research suggests that analyzing amplitude modulation spectra (Chang et al., 2024; Ding et al., 2017; Durojaye et al., 2021) or spectro-temporal modulation spectra (Albouy et al., 2024; Anikin et al., 2023) provides a parsimonious way to characterize these different categories of acoustic signals. However, drawing general conclusions from such studies is challenging due to variations in the acoustic materials and methodologies used and limited types of speech-song samples analyzed. To address some of these limitations, we present to the research community CoVox, a newly developed, fully matched dataset of contrasting vocalizations.

CoVox features the same voices (22 versatile classical singers) performing the same material in three distinct singing styles (as a lullaby, as a pop song, and as an opera aria) or speaking the corresponding text aloud in two speaking styles (as if speaking to an adult or an infant). The five vocalization styles were chosen as a subset of all possible categories of human vocalizations, sampled from a multidimensional continuum – e.g., the speech–music continuum proposed by Phillips (2023 – inspired by Brown, 2000). Please see Fig. 1 for an illustration of the tentative positions of the vocalization categories of the CoVox dataset on the semanticity/discreteness dimensional space reported by Phillips (2023). While the *x*-axis represents how structural features, especially discreteness in pitch and time, increase from speech to music (i.e., music has discrete tones in terms of pitch and duration), the *y*-axis represents how semanticity (i.e., meaning) increases from music to speech. The three song (sub)categories (i.e., pop, lullaby, and opera) would be placed within the song category, but their relative positions are open to interpretation. Our proposed adult-directed speech condition resembles declamation or heightened speech, and the infant-directed speech condition stands between song and adult-directed speech.

**Fig. 1:**
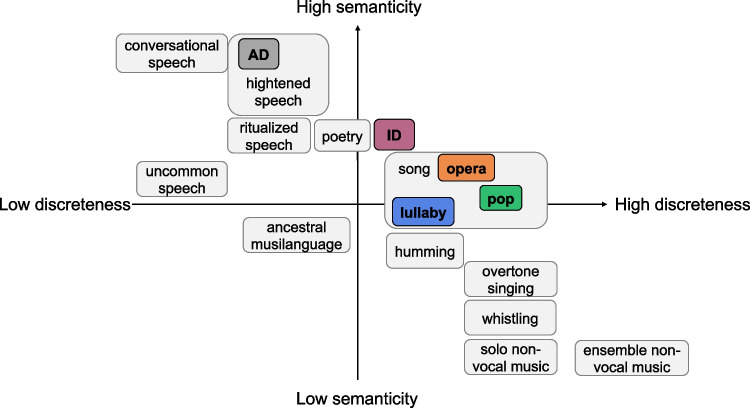
Illustration of the categories of vocalization included in the CoVox dataset and their tentative positions in the speech–music continuum. *Note.* The *x*-axis represents how structural features, especially discreteness in pitch and time, increase from speech to music. The *y*-axis represents how semanticity increases from music to speech. The three song categories are nested within a broader ‘song’ category, though their relative positions on the axes are open to interpretation. The CoVox adult-directed speech condition involves speaking the songs’ lyrics out loud, thus resembling heightened speech more than conversational speech. Adapted with permission from Phillips (2023). *AD* adult-directed speech, *ID* infant-directed speech

Existing datasets often include one single language (e.g., Chong et al., 2023; Livingstone & Russo, 2018) or multiple languages (Albouy et al., 2024; Anikin et al., 2023; Ding et al., 2017; Ozaki et al., 2024). While there are advantages and disadvantages to both approaches, we focus exclusively on one language (Brazilian Portuguese), and extend the range of performances by introducing an additional option: performances without semantic content. The melodies were performed both with the original lyrics and with a/lu/sound, in a condition that closely mirrored the prosodic characteristics from performances with lyrics (as supported by acoustic analyses). Note that the semantic content of performances with lyrics can also be considered unintelligible for speakers unfamiliar with Portuguese and closely related languages (but could still potentially partially convey meaning). Importantly, we recorded multiple singers in five contrasting styles of vocalizations and built a fully matched dataset. Previous studies have recorded contrasting singing styles from one singular, versatile performer (e.g., Stone et al., 2003; Sundberg et al., 1993; Thalen & Sundberg, 2001). Whereas such an approach allows an in-depth analysis of the different styles by one singer, our approach is appealing given that vocalizations are highly variable both within and between individuals (Lee et al., 2019) and that this variability affects the perception of voices and speech (Lavan et al., 2019; Liberman et al., 1967; Weatherholtz & Jaeger, 2015). A fully matched dataset may help to disentangle these sources of variation at both the production (e.g., by close examination of acoustic characteristics) and the perception levels (e.g., by using performances from CoVox as stimuli in perceptual experiments). CoVox is thus a flexible resource with multiple use possibilities.

In the following sections, we first detail the creation of the CoVox dataset. Next, we discuss its validation through a behavioral experiment, demonstrating that lay listeners can accurately recognize the intended vocalization styles. Finally, we present the results of acoustic analyses, which reveal clear contrasts between performances in different styles.

## Creation of the dataset

### Method

#### Singer participants

Twenty-two highly trained Brazilian female classical singers (16 sopranos, six mezzo-sopranos, with ages ranging from 22 to 51 years, *M* = 32.5, *SD* = 7.1) participated in the recording sessions. Recruitment was conducted through personal contacts. Singers reported classical singing training ranging from 4.5 to 27 years (*M* = 12.9 years, *SD* = 6), as well as experience in diverse musical genres, including pop, Música Popular Brasileira (MPB, a genre of popular Brazilian music), jazz, gospel, and musical theatre. At the time of participation, singers reported being engaged in singing activities for durations ranging from one to 40 h/week (*M* = 15.9 h, *SD* = 9.9, including practice time). They also reported a range of solo and ensemble performance activity, with five exclusively performing as soloists, five predominantly as soloists (75% solo, 25% choir), five equally dividing their time between solo and ensemble singing, and seven primarily engaging in ensemble singing (25% solo, 75% choir). Furthermore, singers disclosed commencing voice lessons between the ages of 6 and 25 years old (*M* = 17.7 years, *SD* = 5.7), accumulating music training of between 4 and 30 years (*M* = 15.7, *SD* = 7.3), and playing musical instruments for periods ranging from 0 to 15 years (*M* = 4.5 years, *SD* = 3.9).

#### Material

The melody excerpts correspond to the initial phrases of six Brazilian songs. Four of them are widely known Brazilian folk songs: the lullabies Nana Nenê and Boi da Cara Preta, and the play songs Alecrim and Nesta Rua. We also selected one song from popular Brazilian music repertoire (Música Popular Brasileira, MPB), Chove Chuva, by Brazilian artist Jorge Ben Jor (1939–), and the art song Melodia Sentimental, from the symphonic poem A Floresta do Amazonas by Brazilian classical composer Heitor Villa-Lobos (1887–1959), featuring text by Dora Vasconcellos (1910–1973). Singers received sheet music well in advance of their scheduled recording session to ensure thorough preparation (most singers received sheet music 3–4 weeks prior to their recording session; four singers were last-minute substitutes due to cancellations but received sheet music at least 2 days before their recording session). Please see Supplementary Fig. 1 for sheet music and Supporting Text S1 for translations and syllable segmentation of the texts from the melody excerpts.

#### Procedure

##### Recording sessions

Singers were invited to participate in recording sessions lasting approximately 1 h, conducted in a professional music recording studio situated in Sao Paulo, Brazil, during March 2022. Recordings were made using a microphone in cardioid pattern, and using the Mac standard for lossless audio, with 24 bits per sample and 44.1-kHz sampling rate (Audio Interchange Format, AIFF). A standardized recording protocol was maintained consistently across all sessions. Singers were instructed to stand on predetermined marks on the floor. The microphone-to-mouth distance was maintained at approximately 10 cm for lullaby performances, 30–40 cm for pop renditions, and 60 cm for operatic performances. A recording technician additionally adjusted the gain of the microphone for performances in different styles to maintain good signal rates and avoid clipping, since performances varied greatly in terms of sound intensity level. Singers performed all melodies in one style before moving on to the next, while adhering to the following order and instructions:

- for lullaby singing: imagine you have a baby on your chest and you want to make it fall asleep;
- for pop singing: imagine you are performing a pop song using a microphone;
- for operatic singing: imagine you are on stage performing an opera aria;
- for adult-directed speech: read the text of the melody aloud, as if it was the translation of a piece you just performed on stage;
- for infant-directed speech: now read the same text again, but imagine you are talking to an infant.

The starting note of each melody was played on a keyboard before each performance. Operatic singing performances were recorded at a higher pitch than pop and lullaby versions to achieve naturalistic performances and ensure singers’ comfort, considering that classical singing typically has a higher pitch than pop and lullaby singing. Specifically, for all melodies except Melodia Sentimental, operatic singing was recorded one-fifth higher than pop and lullaby singing. The excerpt of Melodia Sentimental was recorded one-fourth higher due to its extensive range, potentially challenging for mezzo-sopranos. Performances involving the/lu/sound were recorded immediately following the corresponding performance with lyrics. The vowel/u/was chosen based on the first author’s experience as a voice teacher, considering its suitability for Brazilian female singers in achieving a consistent and homogeneous sound. For each singer, a total of 60 distinct conditions were recorded (six melodies, across five vocalization styles and two production types), resulting in a total of 1320 performances in the dataset.^1^ A minimum of three takes was recorded for each singing condition, and for each speech condition, a minimum of two takes was recorded. Upon request from the recording technician or the singers, additional takes were occasionally recorded.

Note that two singers reported having acute voice problems at the moment of recording: singer S14 reported having an allergy crisis, and singer S19 mentioned that her voice was “tired”, but as the recording session progressed, her voice was breathier, with occasional voice cracks. We included their recordings here since they add variability and were particularly scrutinized in the validation and acoustic characterization phases, but excluding their performances only slightly changes the proportion of style recognition in the validation experiment.^2^ Singers provided written consent for the sharing of their recordings for non-commercial purposes and in an anonymized way, under a CC BY-NC-SA license, and were paid in local currency.

##### Audio processing and take selection

Individual takes of recorded performances were cut using Audacity software (version 3.1.3). For each singer, one take for each of the 60 conditions was selected by the first author for further analysis, based on the following (admittedly arbitrary) criteria to exclude takes: occasional ambient noise (e.g., coming from singers’ movements), low vocal quality, low expressiveness, and low authenticity. The final set of 1320 stimuli was normalized to ensure a similar loudness level within style. Using the software To Audio Converter (version 1.0.22—1286), stimuli were loudness normalized without any dynamic range compression, following the EBU R128 standard (European Broadcasting Union, 2023). This standard uses an integrated loudness measure (Loudness Units relative to Full Scale, or LUFS), which aligns better with human perception of loudness compared to RMS or peak normalization. For the validation experiment, all operatic stimuli were normalized to –14 LUFS, all pop singing and speech performances to –18 LUFS, and all lullaby stimuli to –25 LUFS. We also conducted a control experiment in which all stimuli were loudness-normalized to the same level of –23 LUFS. Finally, for sharing the dataset, we have normalized all stimuli to the same level of –23 LUFS.

## Validation of the dataset

A behavioral lab experiment was proposed to examine how well lay listeners could recognize the intended style of the vocalizations, that is, the style of singing performances and the directedness of the speech performances.

### Method

#### Participants

Seventy-five participants (43 self-reported as female, 31 as male, one undisclosed; *M* = 47.3 years old, *SD* = 16.9; 67 with German as their mother tongue, from which seven bilinguals; none of whom reported speaking Portuguese) were recruited from the participant database of the Max Planck Institute for Empirical Aesthetics, in Frankfurt, Germany. They were mostly lay listeners and reported not having any hearing impairment. According to an 18-item adapted version^3^ of the scale of music sophistication of the Goldsmiths Music Sophistication Index (Gold-MSI; Müllensiefen et al., 2014), participants’ average music sophistication score was 81.2 (*SD* = 17.7). Participants were randomly assigned to one of three groups, which differed only in terms of which stimuli they were presented with (see details in the Procedure section). Participants were compensated at the rate of 14€ per hour of participation.

##### Rationale for the sample size

We conducted a power analysis using the function pwr.p.test from the pwr package in R (Champely, 2020). Our analysis plan consisted of using *Z*-tests of proportion to compare the proportion of correct style recognition to chance-level performance (33% for singing performances and 50% for speech performances). The power analysis showed that keeping alpha at the standard 0.05 value and aiming for power of 0.8, a sample size of 24 participants would be sufficient to detect a moderate effect size of h = 0.51 (corresponding to the difference between a proportion of 68% correct recognition and chance level performance at 33%) for the singing stimuli; and that a sample of 23 participants would be sufficient to detect a moderate effect size of h = 0.52 (corresponding to the difference between a proportion of 75% correct recognition compared to chance level performance at 50%) for the speech stimuli. Given the high number of stimuli to be evaluated, we recruited a total of 75 participants, divided into three different groups, so that each stimulus was judged by 25 participants.

#### Material

The stimulus set used in the validation experiment consisted of 1316 vocal performances^1^. All operatic stimuli were normalized to the same loudness level of –14 LUFS, all pop singing and speech performances to –18 LUFS, and all lullaby stimuli to –25 LUFS. These levels were in line with the expected sound pressure level at production (louder for operatic singing, intermediary for pop and speech performances, and quieter for lullabies) and were chosen to allow for comfortable and naturalistic listening of all performances.

#### Procedure

**Fig. 2:**
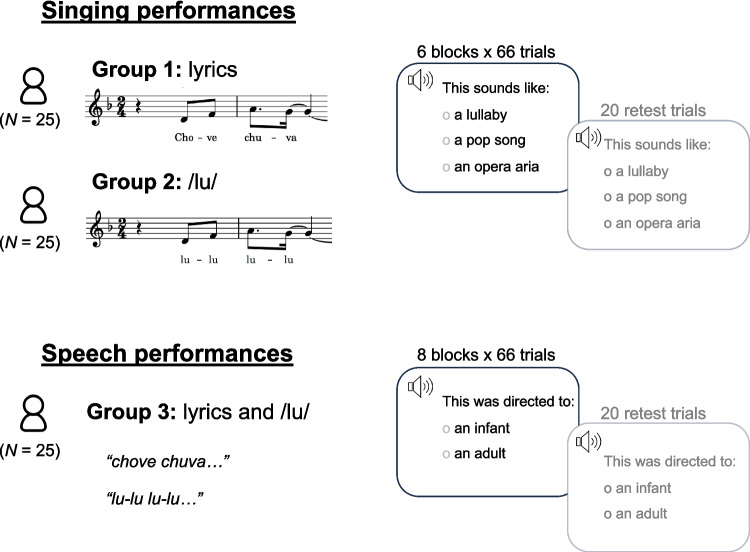
Illustration of the procedure of the validation experiment. *Note.* Each participant was presented with a subset of all performances: only singing performances with lyrics for group 1, only singing performances with /lu/ for group 2, and all speech performances for group 3 (both with lyrics and with/lu/, in a blocked and counterbalanced order). Participants completed a forced-choice task indicating the intended style of singing performances and the intended directedness of speech performances

The experiment was implemented in Labvanced (Finger et al., 2017). The experimental procedure was ethically approved by the Ethics Council of the Max Planck Society and conducted with the written informed consent of each participant. Participants were tested in the laboratories of the Max Planck Institute for Empirical Aesthetics, in Frankfurt, Germany. Please see Fig. 2 for an illustration of the experimental procedure.

The experimental session began with oral and written instructions, which were followed by four practice trials using example stimuli (which were not part of the final stimulus set), presented through headphones (Beyerdynamic DT 770 PRO 80 Ohm) at a volume adjusted to a comfortable level. Participants were assigned to different groups (Fig. 2). For each group, stimuli from different melodies and styles were presented intermixed and in random order. The experimental task was a forced-choice task. In each trial, participants had to indicate if a given stimulus sounded like a lullaby, a pop song, or an opera aria (groups 1 and 2), or if it was directed to an adult or an infant (group 3). The visual display of response alternatives was presented in a fixed order for each participant across the whole session, but this order was counterbalanced across participants. For groups 1 and 2, the experiment was divided into six blocks of 66 trials (except for the last block, which was slightly shorter due to one or three missing stimuli^1^), for a total of 395 or 393 performances, respectively. For group 3, the experiment was divided into eight blocks of 66 trials, for a total of 528 performances. Participants could take a break between blocks. The testing session lasted between 85 and 120 min. Each stimulus was presented once, except for 20 repetitions of a random subset of stimuli (different for each participant), which were used to estimate the test–retest intra-rater agreement. For groups 1 and 2, these 20 repetitions were presented after the last block of trials. For group 3, ten repetitions were presented after the last block with lyrics stimuli, and ten repetitions after the last block with/lu/stimuli. At the end of the session, participants completed the adapted version of the general music sophistication scale of the Goldsmiths Music Sophistication Index (Müllensiefen et al., 2014).

### Statistical analyses

The validation experiments’ preregistration can be found at https://osf.io/wuvb8. All analyses were performed using R Statistical Software (version 4.1.2; R Core Team, 2021) and R Studio (version 2022.7.1.554; RStudio Team, 2022).

#### Analysis of intra-rater agreement

We calculated the test–retest intra-rater agreement based on a subset of 20 repeated trials (Fig. 2). Cohens’ kappa was calculated using the kappa2 function from the irr package in R (Gamer et al., 2019). These values were computed separately for groups 1, 2, and 3. Note that due to a mistake in the coding of the experiment, for a subset of ten participants of group 2, the planned repeated trials were not in fact repeated trials, but “new” stimuli with lyrics instead of /lu/. Because of this, computation of kappa for group 2 is based only on the 15 participants that were correctly presented with repeated trials.

#### Analysis of accuracy of style recognition

To test if the different vocalization styles were recognized, we compared the proportion of correct responses across all participants in each style against chance level performance (33% for singing styles and 50% for directedness of speech), with *Z*-tests for proportions (one-tailed; with the R function prop.test; separately for singing and speech). The reported *p* values are adjusted with the Holm method to control the family-wise error rate (Holm, 1979). Additionally, we also calculated unbiased hit rates for style recognition (Wagner, 1993). Unbiased hit rates were first proposed to account for potential biases in participants’ answers for certain categories in tasks using nonverbal stimuli (e.g., emotion recognition) and are defined as “the joint probability that a stimulus category is correctly identified given that it is presented at all and that a response is correctly used given that it is used at all” (Wagner, 1993, p. 3). To compute unbiased hit rates in the style recognition task, we first built a confusion matrix of intended style as rows and participants’ answers as columns (separately for singing and speech performances). The unbiased hit rate of a particular style was then calculated based on the number of responses in the corresponding cell in the matrix for that style, which was squared and divided by the product of the marginal values of the corresponding row and column. We also followed Wagner (1993) in computing an unbiased chance-level performance estimate as the joint probability of the co-occurrence by chance of a stimulus and response of a corresponding category by multiplying together the independent probabilities of each of these occurring alone (please see accompanying.Rmd files for analyses code). This procedure resulted in an unbiased hit rate (and unbiased chance-level estimate) for the classification accuracy of each vocalization style. As planned in our preregistration, we focus statistical comparisons on the proportion of correct recognition. The additional analyses are descriptive and complementary.

To test if accuracy was similar for performances with lyrics and/lu/, we used two-tailed *Z*-tests for proportions (for singing performances: group 1 vs. group 2; for speech performances: group 3 lyrics vs/lu/). Additionally, for each group, we also compared styles pairwise with *Z*-tests for proportions (two-tailed; adjusting *p* values with the Holm method). Further, to better understand the role of interactions between the performed style, the type of production, and the different melodies, we fit a mixed-effects logistic regression model to predict accuracy of recognition (coded as a binary outcome: 0 = incorrect, 1 = correct response) based on the style of vocalization (adult-directed, infant-directed, lullaby, pop, opera), the type of production (lyrics or/lu/), and the melody. The model included random intercepts for participants, stimulus items, and singers. We used the glmer function from the lme4 R package (Bates et al., 2015), and applied sum contrasts for the categorical predictors Style and Melody (model syntax: Accuracy ~ 0 + Style + Type + Melody + Style:Melody + Style:Type + Type:Melody + (1 | Participant) + (1 | Stimulus) + (1 | Singer), family = binomial(link = “logit”)).^4^

### Results and discussion

Analysis of test–retest intra-rater agreement indicated that participants were (self-)consistent in their responses, with kappa values ranging from 0.59 to 0.73 depending on the group (Supplementary Fig. S2, values within brackets).^5^ This corresponds to between moderate and substantial agreement according to Landis and Koch (1977). Inspection of individual participants’ performances indicated that, with the exception of one participant in group 3, all participants could recognize the intended style of vocalization above chance level, and the majority of participants could do the task with good accuracy (see Supplementary Fig. S2).

**Fig. 3:**
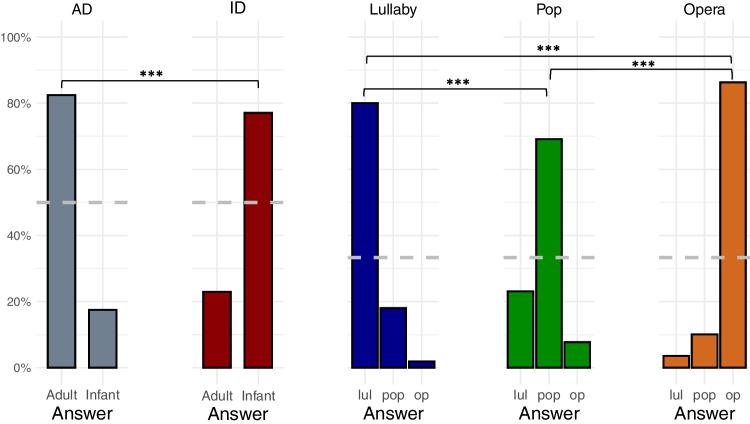
Classification of styles in the validation experiment*. Note.* In each plot, the *y*-axis depicts the proportion of given responses in trials with different styles of vocalization. The *dashed gray horizontal line* represents chance-level performance (33% for singing performances and 50% for speech performances). *AD* adult-directed speech, *ID* infant-directed speech, *lul* lullaby, *op* opera. * *p* < 0.05; ** *p* < 0.01; *** *p* < 0.001

Figure 3 displays the overall accuracy of style recognition. For all styles, the proportion of correct recognition (CR) was higher than chance-level performance (all adj. *p*s < 0.001), ranging between 69.1% (for pop singing) and 86.4% (for operatic singing). Note that unbiased hit rates (Wagner, 1993) were 10.1 to 29% lower than the proportion of CR, as were the unbiased chance-level estimates (see Supplementary Table S1). As displayed in Fig. 3, the majority of mistakes for singing style classification corresponded to participants answering that pop performances were lullabies. Pairwise comparisons of the proportion of CR in each vocalization style (separately for singing and speech performances) showed that recognition was highest of all for operatic singing, followed by lullaby, and lowest for pop singing; and that CR was higher for adult- than infant-directed performances. Supplementary information regarding the proportion of CR by melody, by type of production, and by singer can be found in Supplementary Table S2. The proportion of CR by singer and by style is presented in Supplementary Fig. S3. Finally, Supplementary Fig. S4 presents the proportion of CR by stimulus item.

#### Small differences depending on the type of production and the melody

When comparing the two types of production, we found that correct recognition was higher for performances with lyrics than with/lu/(for singing performances: 81.2% CR for lyrics, 75.8% CR for/lu/; *χ*^*2*^(1) = 82.6, *p* < 0.001; for speech performances: 82% CR for lyrics, 77.5% CR for/lu/, *χ*^*2*^(1) = 42.5, *p* < 0.001). This suggests that, even though participants did not understand the lyrics of the melodies (performed in Brazilian Portuguese), they could still benefit from the phonetic variability of performances with lyrics when identifying the style of singing. An alternative (non-excluding) explanation is that singers were better able to match the target style when singing with lyrics than with/lu/. Note that, at the end of the experiment, the 50 participants of groups 1 and 3 (who were presented with performances with lyrics) were asked if they recognized the language of the performances, to which 20 responded positively – though only 14 correctly specified the language to be Portuguese (or in two cases, Brazilian Portuguese).

#### Interactions between style, type of production, and melody

A mixed effects logistic regression model was proposed to better assess the variability in style recognition across the different conditions in the dataset. The model predicted accuracy on a trial-level from style, type of production, melody, and their two-way interactions, and included random intercepts for subjects, items, and singers. This model revealed main effects of style, type of production, and melody (Supplementary Fig. S5). Please see Supplementary Table S3 for model estimates. Whereas there was no interaction between style and type or melody and type of production, we observed an interaction between style and melody (Supplementary Fig. S5D-F). The latter is particularly interesting since it suggests that melodies were better recognized when performed in a style congruent with their original genre. That is, the melodies Nana Nenê and Boi da Cara Preta, which are originally lullabies, were recognized with highest accuracy when performed as lullabies; the melody Chove Chuva, which is originally a pop (“MPB”) song, was better recognized when performed as a pop song; and the melody Melodia Sentimental, which is originally an art or classical song, was better recognized when performed as an opera aria. It is not clear how much these differences relate to singers’ preconceptions of how such familiar material should be performed (e.g., singing a very well-known lullaby as an opera aria may be challenging), and how much they relate to structural features of the melodies being more appropriate for certain styles of singing, in a “form and function” type of relationship (Mehr et al., 2018; Pisanski et al., 2022). Even though we made an effort to select simple melodies, it may be that, for instance, Melodia Sentimental, with its wider range and sustained notes, was more suitable for classical technique than to be sung as a lullaby, with these structural features “accentuating” the operatic character of the performances.

#### The specific role of loudness

As described above, in the validation experiment we normalized the loudness of the performances to different levels depending on the style. We conducted an additional experiment to investigate if the three singing styles would still be highly recognizable if all stimuli were presented at the same loudness level. We used a subset of three melodies, for a total of 396 singing performances, both with lyrics and/lu/. All stimuli were loudness normalized following the EBU-R128 standard to − 23 LUFS. Ten participants were invited to the laboratory to perform the same singing style recognition task described before. The overall proportion of CR in this control experiment (72.2%) was lower than in the main experiment (79%, *χ*^*2*^(1) = 94.8, *p* < 0.001), but was still above chance level for all styles (all *p*s < 0.001). The slightly higher rate of CR in the main experiment suggests that the difference in loudness levels between styles probably contributed to, but was not essential to, correct style recognition in that experiment. Please see the Supplementary Information (Supporting Text S2, Supplementary Figs. S6, S7, and S8, and Supplementary Table S4) for details, as well as Bruder and Larrouy-Maestri (2023) for a discussion about the role of loudness and other acoustic features in singing style recognition.

## Acoustic characterization of the dataset

In order to characterize the contrasting vocalization styles of the dataset, we conducted acoustic analyses, using computationally-extracted features commonly used to describe speaking and singing voices.

### Methods

#### Acoustic analyses

For all 1320 performances and based on recordings loudness normalized to the same level of –23 LUFS, we used Praat (Boersma, 2001, version 6.0.46) with the settings pitch floor = 75 Hz and pitch ceiling = 800 Hz to extract the following measures: mean and median fundamental frequency (*f*ₒ), its maximum (*f*ₒ max), minimum (*f*ₒ min) and standard deviation (SD of *f*ₒ); shimmer_local (perturbation in the amplitude of *f*ₒ); and jitter_local (perturbation in the periodicity of *f*ₒ). Using VoiceSauce (Shue et al., 2011), with the same settings as in Praat, and also based on whole performances, we extracted the two following measures: Cepstral peak prominence (CPP), a speech quality measure of the relative levels of harmonic and inharmonic energy in the voice, based on the algorithm described by Hillenbrand et al. (1994). CPP is the dB difference between the cepstral peak and a linear regression line measured at the corresponding frequency. Lower CPP values have been perceptually associated with breathiness and dysphonia (Murton et al., 2020). Harmonics-to-noise ratio in the 0–3.5 kHz band (HNR35): corresponds to the ratio between periodic and non-periodic components of the signal, based on the algorithm described by Krom (1993). HNR measurements are obtained by lifting the pitch component of the cepstrum and comparing the energy of the harmonics with the noise floor. Additionally, we estimated the syllable rate of each performance, by dividing the number of syllables in each excerpt by the performance duration in seconds. We also used the analyze function from the Soundgen R package (Anikin, 2019) to batch extract multiple acoustic features (using window length = 20 ms and otherwise default settings). Soundgen is an open-source toolbox for voice synthesis, manipulation, and analysis, originally developed to deal with human nonverbal vocalizations. In our characterization of contrasting vocalizations in the dataset, we focused on four of these features. Harmonic height (harmHeight): estimates how high harmonics reach in the spectrum, that is, the frequency up to which there are still harmonics (or peaks at multiples of *f*ₒ) in the spectrum (in Hz). Harmonic energy (harmEnergy): estimates the amount of energy in upper harmonics, by computing the ratio of total spectral energy above 1.25 × *f*ₒ to the total spectral energy below 1.25 × *f*ₒ (in dB). Depth of amplitude modulation (amEnvDep_mean): estimated from the smoothed sound’s amplitude envelope, this measure quantifies variations in loudness due to amplitude modulation. It ranges from 0 to 1, with higher values indicating more pronounced changes in loudness. Depth of frequency modulation (fmDep_mean, in semitones): a measure of how pronounced is the frequency modulation of a sound. Larger values indicate greater variation in pitch due to frequency modulation. We also make available (but don’t discuss) other features from VoiceSauce, from the Soundgen batch extraction, and from the Essentia toolbox (Bogdanov et al., 2013), an open-source C + + library for music information retrieval (MIR). Please see the Supplementary Information (Supporting Text S3) for details on these additional features. Note that an alternative acoustic analysis of the singing performances, based on individually segmented notes, is described in Bruder and Larrouy-Maestri (2023) and is openly available.

#### Statistical analyses

To explore the dimensionality of the acoustic space of the vocalizations, we conducted a principal component analysis (PCA), using the prcomp function in R. We also conducted an overall comparison of all vocalization styles, by fitting linear mixed models predicting each of the selected 11 acoustic features from the style of vocalization (adult-directed, infant-directed, lullaby, pop, opera), the type of production (lyrics or/lu/), and the melody, and their two-way interactions. We used the lmer function from the lme4 R package (Bates et al., 2015). Random intercepts for singers and random slopes for the effect of vocalization style over singers were included to account for variability between singers. Sum contrasts were applied to the categorical predictors of style and melody. The model syntax was as follows: lme4::lmer(Feature ~ 0 + Style + Type + Melody + Style:Melody + Style:Type + Type:Melody + (1 + Style | Singer)). Mean *f*ₒ values were converted from Hz to semitones with a reference value of 342 Hz (which corresponds to the average *f*ₒ found in lullaby and pop singing), using the formula: *f*_semitones_ = 12(log_2_. *f*_Hz_/342). Other frequency_-_related variables (SD of *f*ₒ_,_ jitter, shimmer, depth of frequency modulation, and harmonic height) were log-scaled to improve model fit. Additionally, all variables (except for mean *f*ₒ) were zero-centered prior to fitting the linear mixed models. Pairwise comparisons for the effect of style were computed using the avg_comparisons function from the *marginaleffects* R package (Arel-Bundock et al., 2024).

### Results and discussion

A total of 1320 vocal performances, consisting of six melody excerpts performed by 22 singers, in five styles of vocalizations and two types of production (with lyrics or a/lu/sound) were acoustically analyzed. Singing performances were on average 9.6 s long (*SD* = 1.2, min = 5.9, max = 13.3 s), and speech performances were on average 5.4 s long (*SD* = 1.1, min = 3.5, max = 10.9 s).

#### Principal component analysis

**Fig. 4:**
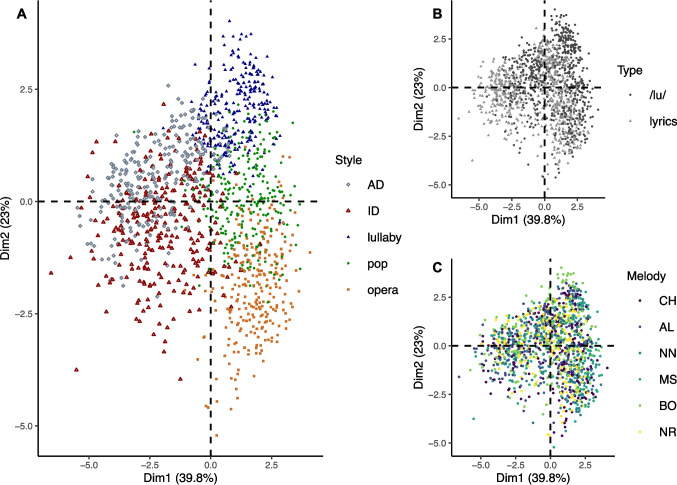
Principal component analysis: Multidimensional visualization of all stimuli by style, type of production, and melody. *Note. N* = 1320 performances. Stimuli are shown as points of different shapes and colors to represent the different: **A** Styles of vocalization (lullaby, pop, or operatic singing; adult- or infant-directed speech); **B** Types of production (/lu/, lyrics); **C** Melodies (*CH* Chove Chuva, *AL* Alecrim Dourado, *NN* Nana Nenê, *MS* Melodia Sentimental, *BO* Boi da Cara Preta, *NR* Nesta Rua). *AD* adult-directed; *ID* infant-directed

The first and second dimensions of the PCA explained 62.8% of the variance, and the first three dimensions combined explained 76.8% of the variance. Figure 4 shows clear clusters for performances in different vocalization styles (Fig. 4A), but not for performances with different types of production (Fig. 4B) or melodies (Fig. 4C). These results are in line with the high proportion of correct style recognition observed in the validation experiment, and support that performances in different styles are acoustically contrasted and distinct, despite being produced by the same singers based on the same melodies. Please see the Supplementary Information for a scree plot and the contribution of each variable to the first, second and third dimensions of the PCA (Supplementary Fig. S9 and Supplementary Table S5). Note that interpretation of the dimensions is not straightforward, with each dimension containing a mix of features associated with *f*ₒ, perturbation/harmonicity, and timbre. Whereas PC1 is associated with perturbation, harmonicity, dynamic loudness changes and syllable rate, PC2 reflects *f*ₒ content (as well as timbral characteristics, with harmHeight and CPP), and PC3 includes breathiness and *f*ₒ content.

#### Acoustic comparisons

**Fig. 5:**
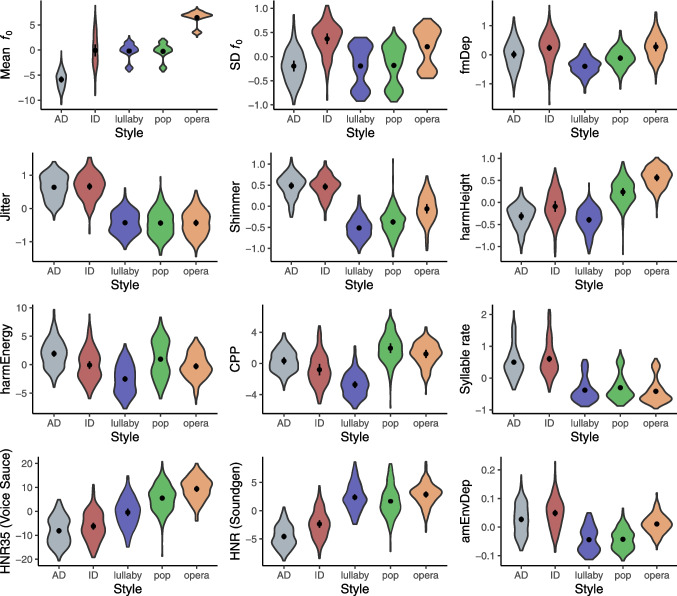
Distribution of acoustic features by vocalization style. *Note. N* = 1320 stimuli (264 per style). Violin plots show the distribution of (zero-centered) acoustic features by style of vocalization. Dots and error bars in the center of each distribution represent model-based estimates and 95% confidence intervals (using the marginaleffects::predictions function). *fₒ* fundamental frequency, *SD* standard deviation, *fmDep* depth of frequency modulation, *harmHeight* harmonic height, *harmEnergy* harmonic energy, *CPP* cepstral peak prominence, *HNR35* harmonics-to-noise ratio between 0 and 3.5 kHz (VoiceSauce). *HNR* harmonics-to-noise ratio (Soundgen), *amDep* depth of amplitude modulation. Please see Supplementary Table S6 for a summary of (untransformed) average raw values for each acoustic feature

**Fig. 6:**
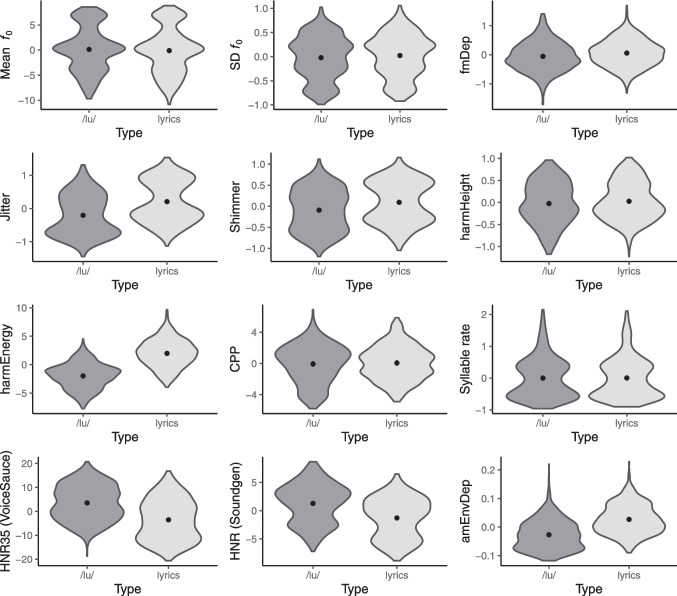
Distribution of acoustic features by type of production. *Note. N* = 1320 performances (660 per type of production). Violin plots show the distribution of (zero-centered) acoustic features by type of production. Dots and error bars in the center of each distribution represent model-based estimates and 95% confidence intervals (using the marginaleffects::predictions function). *fₒ* fundamental frequency, *SD* standard deviation, *fmDep* depth of frequency modulation, *harmHeight* harmonic height, *harmEnergy* harmonic energy, *CPP* cepstral peak prominence, *HNR35* harmonics-to-noise ratio between 0 and 3.5 kHz (VoiceSauce). *HNR* harmonics-to-noise ratio (Soundgen), *amDep* depth of amplitude modulation. Please see Supplementary Table S6 for a summary of untransformed, raw values of each acoustic feature

As expected, the linear mixed models revealed a main effect of style for all acoustic features, supporting that vocalizations are contrasted. Figures 5 and 6 illustrate the distribution of acoustic features by style of vocalization and by type of production, respectively, along with model-based estimates from the proposed linear mixed models. Please see Supplementary Fig. S10 for a similar plot by melody, Supplementary Table S6 for a summary of (untransformed) raw values for each acoustic feature, and Supplementary Fig. S11 for a correlation matrix.

##### Differences between styles of vocalization

In this section, we shortly summarize the most relevant differences in acoustic descriptors by vocalization style. The results of pairwise comparisons of the effect of style for each acoustic feature can be found in Supplementary Table S7.

***fₒ measures***. Mean *f*ₒ values were different from each other for all styles, with the exception of lullaby, pop, and infant-directed speech. In the case of speech performances (for which no starting pitch was given to singers at the moment of recording), we observed differences between infant- and adult-direct speech, with infant-directed speech presenting higher mean *f*ₒ (infant-directed: 350 Hz, *SD* = 67.1; adult-directed: 247 Hz, *SD* = 24.9), higher standard deviation of *f*ₒ (infant-directed: 85.6 Hz, *SD* = 26; adult-directed: 48.9 Hz, *SD* = 17.4) and higher *f*ₒ max–min (infant-directed: 365 Hz, *SD* = 111; adult-directed: 215 Hz, *SD* = 93.8) than adult-directed speech, which is in line with previous observations (e.g., Cox et al., 2022; Cristià, 2010; Fernald & Simon, 1984; Hilton et al., 2022; Narayan & McDermott, 2016; Thanavisuth & Luksaneeyanawin, 1998).

***Jitter and shimmer.*** For both jitter and shimmer, values were higher in speech than in singing performances. But note that these measurements should be interpreted with caution, since they included consonants and were not measured from individual, sustained vowels, as typically reported; and our performances varied simultaneously in different parameters such as *f*ₒ and intensity, all of which could also influence perturbation measures (Brockmann et al., 2008, 2011). Please see Bruder and Larrouy-Maestri (2023) for a comparison of singing styles based on the acoustic analysis of individual notes.

***CPP.*** For CPP, all styles were different from each other. CPP values were the lowest for lullaby singing (*M* = 18.0, *SD* = 1.7), followed by infant-directed speech (*M* = 19.6, *SD* = 2.1), operatic singing (*M* = 20.6, *SD* = 1.1), adult-directed speech (*M* = 21.1, *SD* = 1.4) and highest of all for pop singing (*M* = 22.5, *SD* = 1.5). As a reference for normative values, when measuring CPP with VoiceSauce, Madill et al. (2018) reported average CPP values of 19.2 (*SD* = 1.1) for connected speech and 24.6 (*SD* = 2.8) for sustained/a/vowel (with productions made by female students without voice problems). While our reported values for infant- and adult-directed speech (19.4 and 20.6 for the subset of performances with lyrics) don’t seem different from this norm for connected speech, comparing our singing styles to these two categories of speech is more challenging. Crucially, the singing styles also vary in pitch and sound pressure level, which further complicates such comparisons since these variables may interact with CPP levels (Baker et al., 2022; Brockmann-Bauser et al., 2021). In any case, when comparing CPP values for pop and lullaby performances (which were sung in the same key), the lower values found for lullaby (18 dB) than pop singing (22.5 dB) are likely related to their breathier voice quality (e.g., Murton et al., 2020). Further, our results suggest that the lower CPP values for infant-directed speech (the second lowest of all) are also related to a breathy voice quality, which is in line with previous studies (Cheng et al., 2023; Miyazawa et al., 2017) and supports a role of breathiness as a common vocal quality of infant-directed vocalizations.

***HNR35.*** All styles were different from each other in terms of HNR35. Pairwise comparisons showed that HNR35 values were the highest in operatic singing, followed by pop and lullaby singing, infant-directed speech, and lowest in adult-directed speech. This contrasts with what was reported in Bruder and Larrouy-Maestri (2023), where operatic singing had lower values of HNR35 than the other singing styles, and is likely a consequence of the different loudness normalization levels used for each style (i.e., acoustic analysis was not based on stimuli normalized to the same loudness level).^6^

***Syllable rate.*** All styles differed from each other. Syllable rate was faster for pop than the other singing styles; and faster for speech than singing performances. The latter is in line with previous descriptions of slower temporal rate for singing than speaking (e.g., Ozaki et al., 2024). Syllable rate was also slightly higher in infant- than adult-directed speech, which contrasts with previous findings (e.g., Cristià, 2010; Fernald & Simon, 1984; Narayan & McDermott, 2016; Thanavisuth & Luksaneeyanawin, 1998). However, closer inspection of local temporal variations within performances (for instance as in Martin et al., 2016) may unveil further details in differences between the two conditions.

***Depth of frequency modulation.*** Almost all styles were different from each other, with the exception of adult-directed speech and pop singing, and of infant-directed speech and opera. Values were highest for infant-directed speech and operatic singing. We speculate that the highest values found in operatic singing relate to the use of vibrato, a periodic oscillation in the vocal signals’ *f*ₒ (not analyzed in the present study, but examined in Bruder & Larrouy-Maestri, 2023).

***Depth of amplitude modulation.*** All styles differed from each other, with the exception of lullaby and pop singing. These had the lowest values of all, followed by operatic singing, adult-directed speech, and infant-directed speech. The highest depth of amplitude modulation in infant-directed speech (i.e., more pronounced changes in loudness) agrees with the idea of infant-directed speech as a more dynamic type of vocalization than adult-directed speech not only in terms of pitch and pitch variation, but also in terms of loudness variations.

##### Differences between types of production

Some of the acoustic features also varied depending on the type of production. As illustrated in Fig. 6, values were higher for performances with lyrics than with/lu/in the case of jitter and shimmer, harmonic energy, and depth of amplitude modulation. On the other hand, vocalizations with lyrics exhibited lower values of HNR35 than vocalizations with a /lu/ sound, which is likely related to the vowel variability in performances with lyrics (since HNR values have been described to be higher for /u/ than for /a/ and /i/ vowels—e.g., Teixeira & Fernandes, 2014), as well as due to articulatory differences in terms of consonants. Interestingly, there was no effect of type of production for syllable rate, which suggests that /lu/ performances may be an attractive option for researchers looking for controlled speech (or speech-like) stimuli which preserve important prosodic characteristics without carrying semantic or phonetic information. Note that the performances with lyrics do vary in phonetic content but may also be considered as jabberwocky to participants unfamiliar with Portuguese.

##### Differences between melodies

As illustrated in Supplementary Fig. S10, there were also differences between melodies in all acoustic features, especially for mean *f*ₒ, SD of *f*ₒ, and syllable rate. This is not surprising, considering that melodies typically vary in pitch, pitch range, length, and lyrics. In other words, the structural differences between melodies expectedly led to such acoustic differences. Please refer to our online supporting files (.Rmd and corresponding.html files) for full reporting of the acoustic models.

### Limitations of the acoustic analyses

The acoustic analyses clearly show that vocalizations in different styles are contrasted, but this approach has limitations. For instance, we varied microphone distance (without calibration) and adjusted microphone gain to ensure good signal levels, while avoiding clipping during the recording, which is not the usual practice in the field of voice science (e.g., Švec & Granqvist, 2010, 2018). Also, for the validation experiment, we performed loudness normalization of stimuli to different loudness levels depending on the style (in line with the expected sound pressure level at production) to arbitrary levels, which allowed for comfortable listening of all performances but limits the extent of inference that can be made about differential voice production mechanisms between styles. The acoustic analyses provided here are primarily intended to describe the acoustic signal. Our description of the vocal performances can be expanded in future research to include a broader range of acoustic features (e.g., additional spectral characteristics; detailed comparisons of formants and vowel space area; description of differential spectro-temporal modulation patterns). This would allow for a much finer level of detail and deeper understanding of the vocal performances.

### General discussion

The CoVox dataset features contrasting singing and speaking performances by 22 versatile female classical singers and is freely available to the research community. The validation experiment supports that vocalizations were contrasted enough for lay listeners to recognize the intended style of singing performances and the intended directedness of speech performances with good accuracy, even when stimuli were loudness normalized to the same loudness level (though note that this control experiment was conducted with half of the stimulus set and fewer participants). The differences illustrated by the clear clusters in the two-dimensional acoustic space (Fig. 4) support the expected contrasts between the vocalization styles, which makes CoVox a reliable and versatile source of material to investigate different aspects of human vocalizations.

The acoustic profiles described in CoVox generally match descriptions in the literature (Butte et al., 2009; Cheng et al., 2023; Cox et al., 2022; Hilton et al., 2022; Larrouy-Maestri et al., 2014; Livingstone et al., 2013; Miyazawa et al., 2017; Ozaki et al., 2024). For example, speech performances were faster and had lower pitch than singing performances. Infant-directed speech had higher pitch and wider pitch range, and higher depth of frequency and amplitude modulation, than adult-directed speech. Operatic singing had higher depth of frequency modulation and higher harmonics-to-noise ratio (as well as intense use of vibrato, as described in Bruder & Larrouy-Maestri, 2023). Also, pop singing performances were faster than lullaby and operatic singing performances. In addition to generally matching the acoustic profiles described in the literature, CoVox revealed that both lullabies and infant-directed speech exhibited a breathier voice quality, which provides valuable insight into performance factors related to infant-directedness.

The different vocalization styles included in CoVox may be seen as sampled from different positions in the speech-music continuum (Phillips, 2023) and appeal to researchers looking for singing and speaking stimuli, for instance for perception and/or neuroimaging studies, in which fully matched, controlled, and naturalistic stimuli are desirable. The performances with lyrics, spoken and sung in Brazilian Portuguese, can be used as material not carrying semantic information to non-Portuguese speakers. On the other hand, performances with/lu/, which followed the prosodic profile of corresponding performances with lyrics, may offer an alternative without semantic information and with limited phonetic content. Interested researchers may choose to subset performances by style, by melody, by type of production (with lyrics or with a /lu/ sound), and by singer, or based on the proportion of correct recognition in the validation experiment. In this sense, CoVox allows for more flexibility in comparison to the usual speech versus singing comparison: researchers can contrapose, for instance, adult- versus infant-directed speech; or adult- versus infant-directed performances in general (e.g., grouping adult-directed speech with pop singing; and infant-directed speech with lullaby). Researchers can also focus comparisons on the singing styles, as we have done in Bruder and Larrouy-Maestri (2023). Future research is encouraged to further explore the acoustic characteristics presented and discussed here, as well as the extended set of audio descriptors that we make freely available, along with raw data from the validation experiment.

It is important to mention some limitations of CoVox. First, as noted previously, our recording procedure allows limited insights into differential mechanisms of voice production, and the acoustic analyses reported here only aims to confirm that vocalizations were contrasted. We focused on a core set of 11 relatively easy-to-interpret acoustic features commonly used to describe voices in a “simple but fair” comparison, but other approaches would be interesting to reveal further similarities and differences between the five vocalization styles. Second, our infant-directed performances were simulated. We asked the singers to imagine that they were speaking to an infant (for infant-directed speech) and that they had a baby on their chest and were trying to make it fall asleep (for lullaby singing). As a consequence, the infant-directed performances may sound less contrasting to adult-directed speech than authentic ones, where an infant is actually present (Fernald & Simon, 1984; Trainor, 1996; Trainor et al., 1997). Relatedly, we did not collect personal information on singers’ maternal status. However, Fernald and Simon (1984) reported similar prosodic modifications in infant-directed speech from primiparous and multiparous women, which suggests that extensive previous experience with infants is not a prerequisite for this behavior. Despite these limitations, the high accuracy of style recognition by lay listeners and the observed acoustic contrasts support CoVox as an attractive compromise between controlled and naturalistic-sounding performances, making it a valuable resource for advancing research in human vocal communication.

## Supplementary Information

Below is the link to the electronic supplementary material.

Supplementary file1 (PDF 1.45 MB)

## Data Availability

The CoVox dataset is freely available under a Creative Commons license. The CoVox dataset, as well as the raw data from both validation experiments, can be found at https://osf.io/cgexn/. The main validation experiment was preregistered (https://osf.io/wuvb8).

## Footnotes

1 Unfortunately, nine performances were missing due to technical issues during the recording sessions (S01_NR_pop_U, S01_BO_pop_U, S05_AL_cl_L, S05_AL_cl_U, S07_NR_pop_L, S07_NR_pop_U, S09_NR_pop_L, S09_NR_pop_U; S11_NN_cl_U) but were replaced by additional recordings of good quality sent afterward by the cooperative singers, who recorded themselves a cappella in private settings. Note that 788 of them (395 with lyrics and 393 with/lu/) were used in the validation phase, since four additional recordings were only received after experimental data collection.

2 After exclusion of these two singers, the proportion of correct style recognition changes from 82.2% to 83.1% for adult-directed speech, from 76.8% to 78.6% for infant-directed speech, from 80% to 80.2% for lullaby singing, from 86.4% to 89.3% for operatic singing and from 69.1% to 68.6% for pop singing.

3 We included the following items: AE_01, AE_02, AE_05, AE_07, EM_04, MT_03, MT_07, PA_01, PA_04, PA_06, PA_07, PA_08, SA_01, SA_02, SA_03, SA_04, SA_05, SA_06. These correspond to 15 items from the general sophistication scale, plus three items corresponding to a short scale of perceptual abilities.

4 This model differs from the one proposed in the preregistration (Accuracy ~ Style + Style:Melody + Style:Singer + (1|Participant) + (1|Melody/Singer). We realized that the initial one was less informative than this one for describing the dataset.

5 Note that Krippendorff’s α leads to identical values in this case.

6 Note that we also make available Soundgen’s HNR_mean feature, which, when extracted with a short-enough window length of 20 ms, leads to similar results (i.e., higher values of HNR for operatic singing than for all other styles (please see Fig. 5). Also note that VoiceSauce’s HNR35_mean and Soundgen’s HNR_mean estimates are highly correlated, *r*(1318) =.84, *p* <.001.

## Code availability

Analyses code can be found at https://osf.io/cgexn/
