# Correction: CoVox: A dataset of contrasting vocalizations

**DOI:** 10.3758/s13428-025-02687-2

**Published:** 2025-05-06

**Authors:** Camila Bruder, Pauline Larrouy-Maestri

**Affiliations:** 1 Max Planck Institute for Empirical Aesthetics, Grüneburgweg 14, 60322 Frankfurt Am Main, Germany; 2 Center for Language, Music, and Emotion (CLaME), New York, NY USA

**Correction: Behavior Research Methods (2025) 57:142**

10.3758/s13428-025-02664-9

The original online version of this article was revised: Fig. 3 was mistakenly not updated as requested by the author in the original version of the article. Figure 3 has been updated.

Incorrect Figure 3

**Fig. 3:**
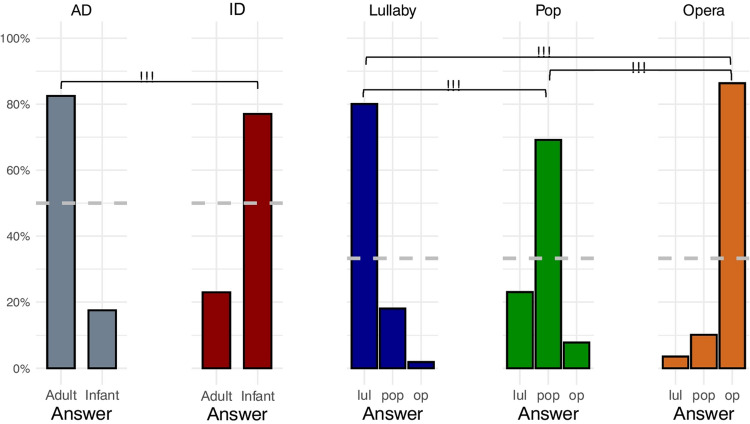
Classification of styles in the validation experiment. *Note*. In each plot, the y-axis depicts the proportion of given responses in trials with different styles of vocalization. The *dashed gray horizontal line* represents chance-level performance (33% for singing performances and 50% for speech performances). *AD* adult-directed speech, *ID* infant-directed speech, *lul* lullaby, op opera. * *p* < 0.05; ** *p* < 0.01;*** *p* < 0.001

Correct Figure 3

**Fig. 3:**
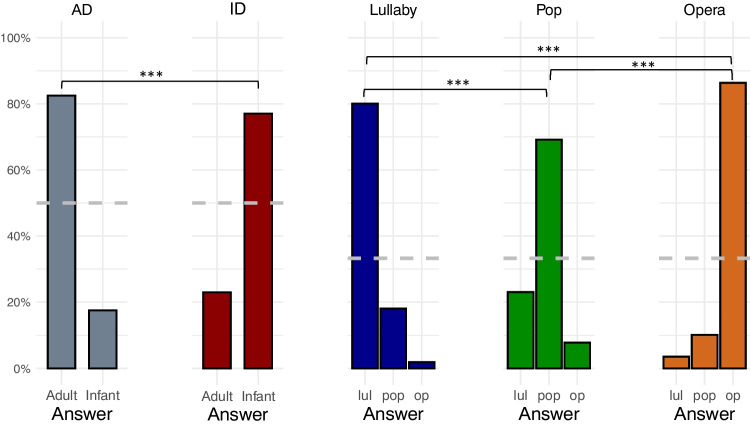
Classification of styles in the validation experiment. *Note*. In each plot, the y-axis depicts the proportion of given responses in trials with different styles of vocalization. The *dashed gray horizontal line* represents chance-level performance (33% for singing performances and 50% for speech performances). *AD* adult-directed speech, *ID* infant-directed speech, *lul* lullaby, op opera. * *p* < 0.05; ** *p* < 0.01;*** *p *< 0.001

