# Supplementary material for: CoVox: A dataset of contrasting vocalizations
Source: Behav Res Methods. 2025 Apr 11;57(5):142. doi: 10.3758/s13428-025-02664-9 (PMC11991967; doi:10.3758/s13428-025-02664-9)
Supplement: Supplementary file 1 — Supplementary file1 (PDF 1.45 MB) [file 13428_2025_2664_MOESM1_ESM.pdf]

Supplementary Materials

CoVox: a dataset of contrasting vocalizations

Bruder, Camila*; Larrouy-Maestri, Pauline*

*** Correspondence:** cajmila@gmail.com

plm@ae.mpg.de

The **CoVox** dataset is freely available under a Creative Commons license.

Please find the CoVox dataset, as well as raw data from the validation experiments and analysis code at https://osf.io/cgexn

Supplementary Figure S1

Musical Notation for the Recorded Melody Excerpts

Nana Nenê (anonymous)

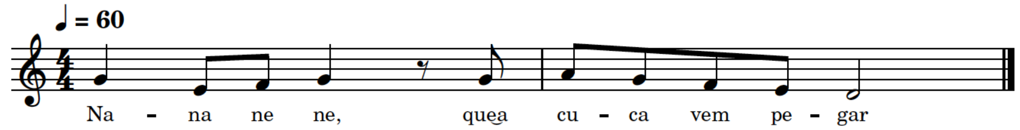

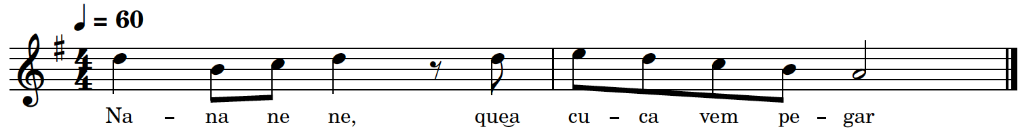

Boi da Cara Preta (anonymous)

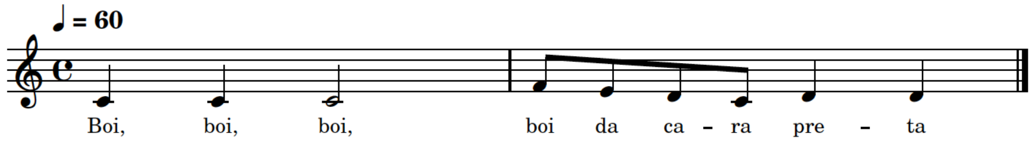

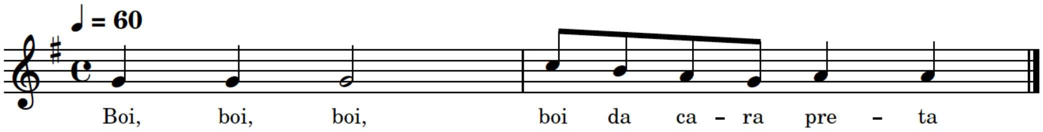

Alecrim Dourado (anonymous)

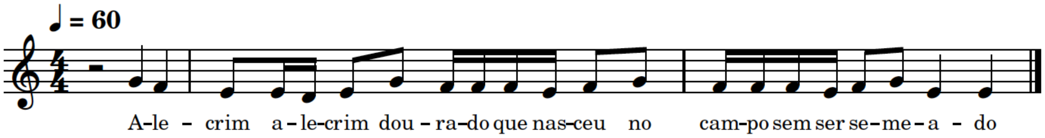

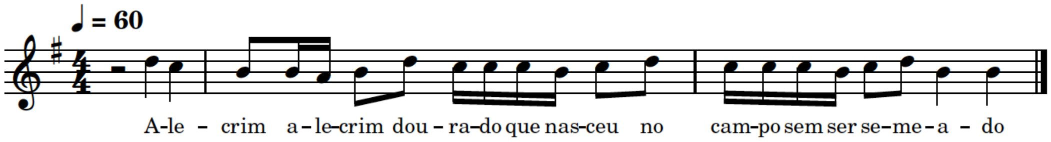

Nesta Rua (anonymous)

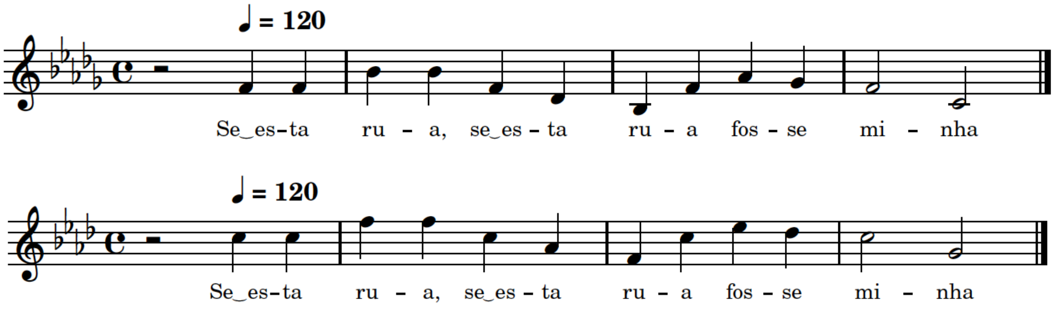

Chove Chuva (Jorge Ben Jor)

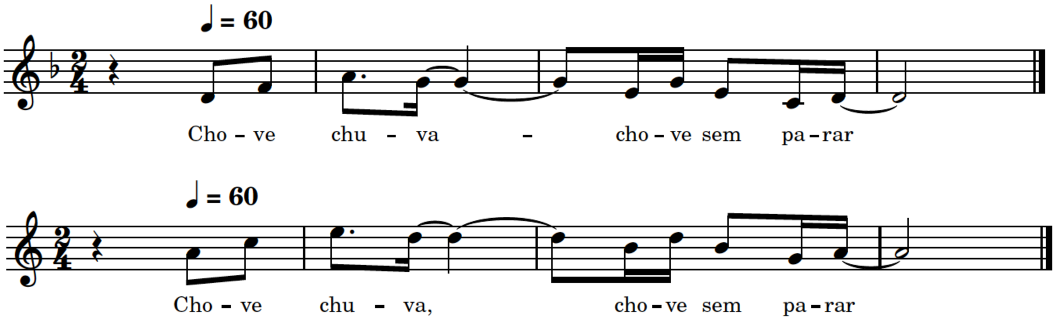

Melodia Sentimental (Heitor Villa-Lobos)

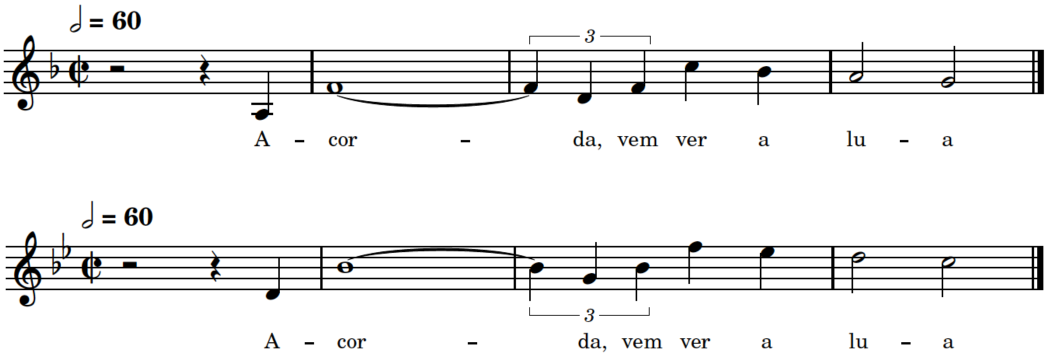

Note. The first version of each melody was used for pop and lullaby versions and the second, transposed a fourth or fifth higher, for the operatic version.

Supporting Text S1

Syllable Segmentation (Determined by Sheet Music) and Translation of the Texts of Melody Material

Nana nenê, que a cuca vem pegar
Na | na | ne | nê | que-a | cu | ca | vem | pe | gar (10 sung syllables)
Sleep, baby, (or) the Cuca will come get

Boi, boi, boi, boi da cara preta
Boi | boi | boi | boi | da | ca | ra | pre | ta (9 sung syllables)
Ox, ox, ox, black-faced ox

Alecrim, alecrim dourado que nasceu no campo sem ser semeado
A | le | crim | a | le | crim | dou | ra | do | que | na | sceu | no | cam | po | sem | ser | se | me | a | do (21 sung syllables)
Rosemary, golden rosemary that was born in the field without being sown

Se esta rua, se esta rua fosse minha
Se-es | ta | ru | a | se-es | ta | ru | a | fo | sse | mi | nha (12 sung syllables)
If this street, if this street were mine

Chove chuva, chove sem parar
Cho | ve | chu | va | cho | ve | sem | pa | rar (9 sung syllables)
It's raining rain, raining non-stop

Acorda, vem ver a lua
A | co | rda | vem | ver | a | lu | a (8 sung syllables)
Wake up, come see the moon

Supplementary Figure S2

Proportion of Correct Recognition by Participant

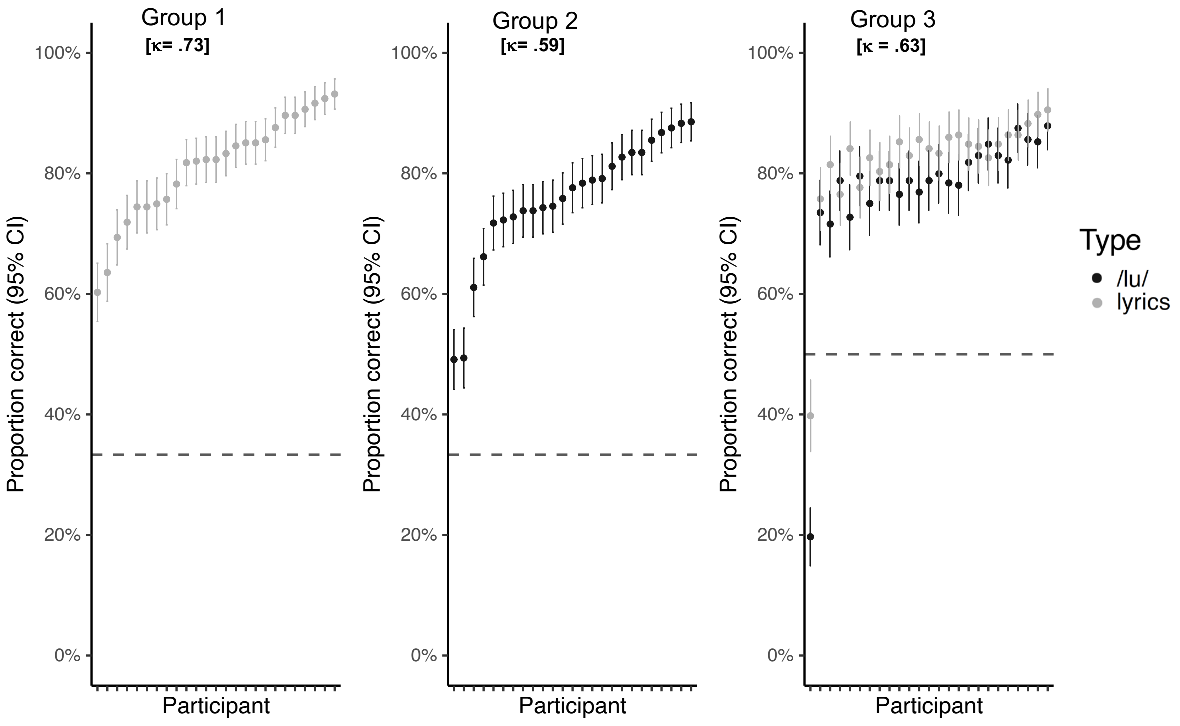

Note. N = 25 participants in each group. Shown are raw percentages of correct recognition for each group of participants. Error bars depict 95% confidence intervals. The dashed gray horizontal line represents chance-level performance. The values between brackets represent intrarater test-retest agreement (at the group level) as measured by Cohens' kappa.

Supplementary Table S1

Proportion of Correct Recognition and Unbiased Hit Rate in the Validation Experiment by Vocalization Style (as %)

Style	PC	*SD*	Chance	Hu	Hu chance
Adult-directed speech	82.2	38.2	50.0	64.5	26.4
Infant-directed speech	76.8	42.2	50.0	62.8	23.6
Lullaby	80.0	40.0	33.3	60.2	12.0
Pop	69.1	46.2	33.3	49.1	10.8
Opera	86.4	34.3	33.3	77.7	10.6

Note. All values shown as percentages. PC: (raw) proportion of correct recognition; SD: standard deviation of PC; Chance: chance-level performance without any correction for bias; Hu: unbiased hit rate according to Wagner (1993). Hu chance: (corrected) unbiased chance-level performance according to Wagner (1993).

Supplementary Table S2

Proportion of Correct Recognition in the Validation Experiment by Melody, Type of Production, and Singer

**Melody**	**% Correct**	***SD***
Boi da cara preta	77.0	42.1
Nesta rua	77.1	42.0
Melodia sentimental	78.2	41.3
Chove chuva	80.3	39.8
Nana nenê	80.3	39.8
Alecrim dourado	80.5	39.6

**Type of production**	**% Correct**	***SD***
/lu/	76.5	42.4
Lyrics	81.4	38.9

**Singer**	**% Correct**	***SD***
S02	63.6	48.1
S14	65.2	47.7
S07	69.8	45.9
S19	72.2	44.8
S18	74.2	43.8
S08	76.1	42.7
S20	76.7	42.3
S03	78.2	41.3
S04	78.2	41.3
S09	78.3	41.2
S16	80.0	40.0
S12	81.0	39.2
S06	82.1	38.4
S11	82.2	38.3

S10	82.3	38.2
S17	82.3	38.2
S21	82.9	37.7
S22	83.3	37.3
S05	83.4	37.2
S15	87.6	32.9
S13	88.6	31.7
S01	88.7	31.6

Note. Shown are raw percentages of correct recognition.

Supplementary Figure S3

Proportion of Correct Recognition by Singer and by Style

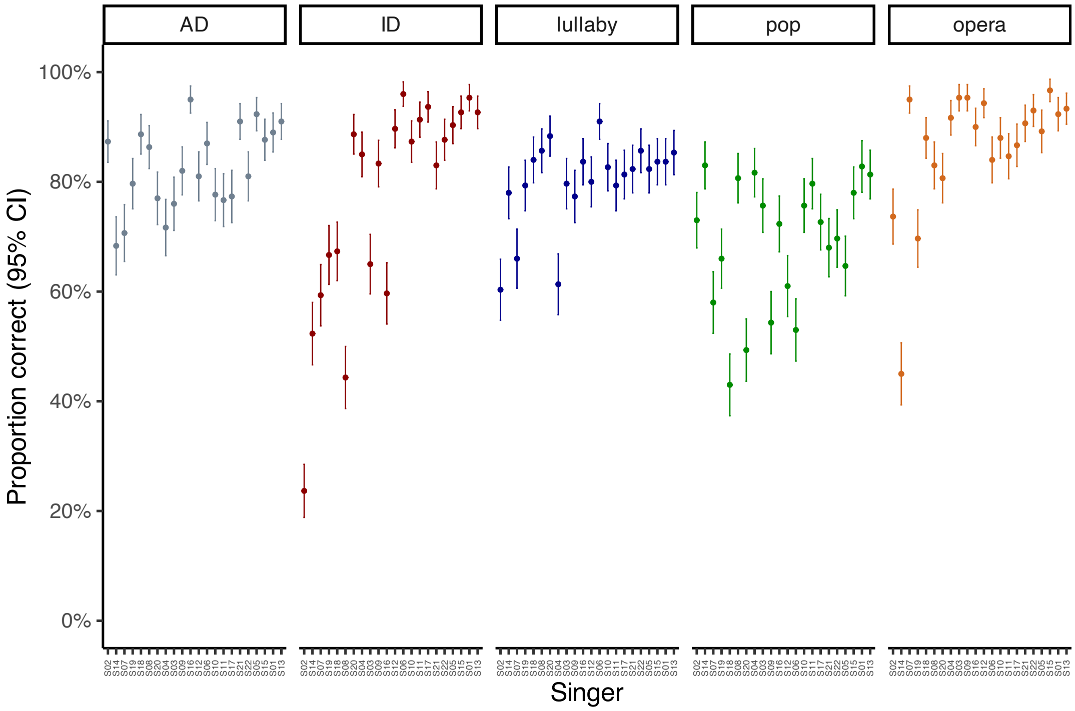

Note. N = 12 performances in each style for each singer (6 melodies x 2 types of production). Shown are raw percentages of correct recognition. Error bars depict 95% confidence intervals. In all facets, singers are presented in the same order, based on the overall proportion of correct recognition across the five styles. AD: adult-directed speech; ID: infant-directed speech.

Supplementary Figure S4

Proportion of Correct Recognition by Stimulus Item

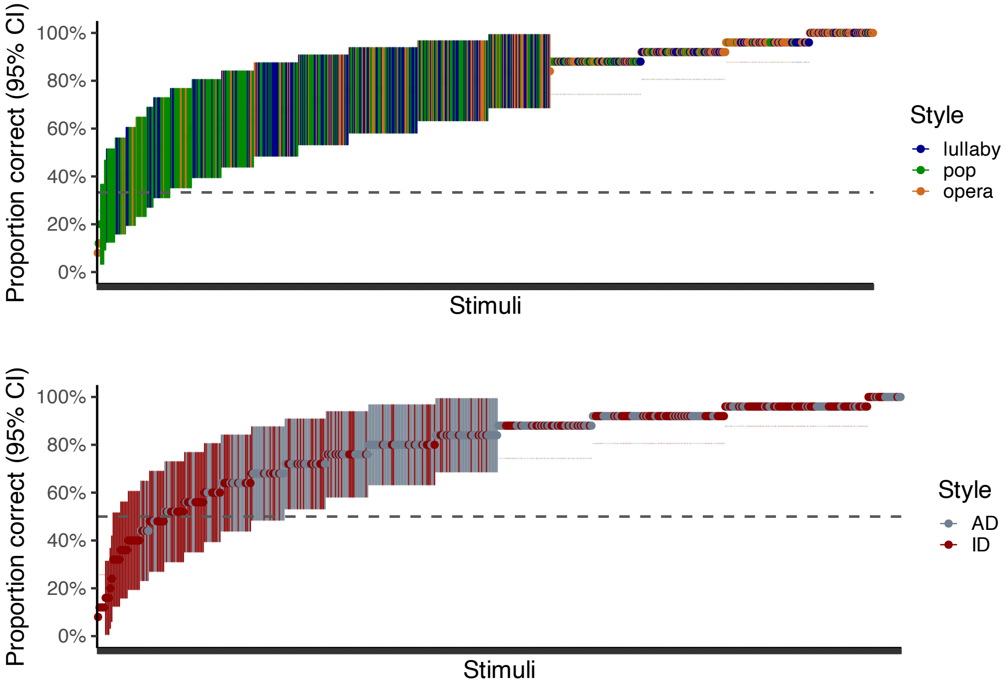

Note. N = 788 singing stimuli (top) and 528 speech stimuli (bottom). Error bars depict 95% confidence intervals. Shown are raw percentages of correct recognition. The dashed gray horizontal line represents chance-level performance. AD: adult-directed speech; ID: infant-directed speech.

Supplementary Table S3

Mixed Effects Logistic Regression of Accuracy of Style Recognition

Predictors	*Log-Odds*	*CI*	*Statistic*	*p*
Style c [AD]	1.68	1.31 – 2.04	8.98	**<0.001**
Style c [ID]	1.41	1.05 – 1.78	7.57	**<0.001**
Style c [lullaby]	1.62	1.25 – 1.98	8.67	**<0.001**
Style c [pop]	0.75	0.39 – 1.11	4.07	**<0.001**
Style c [opera]	2.13	1.76 – 2.50	11.26	**<0.001**
Type [lyrics]	0.45	0.20 – 0.69	3.56	**<0.001**
Melody c1	0.04	-0.13 – 0.21	0.48	0.629
Melody c2	0.07	-0.10 – 0.24	0.79	0.432
Melody c3	0.21	0.04 – 0.39	2.41	**0.016**
Melody c4	-0.03	-0.21 – 0.14	-0.40	0.690
Melody c5	-0.18	-0.35 – -0.01	-2.03	**0.042**
Style c1 × Melody c1	0.29	0.04 – 0.55	2.29	**0.022**
Style c2 × Melody c1	-0.11	-0.36 – 0.13	-0.90	0.370
Style c3 × Melody c1	-0.92	-1.15 – -0.68	-7.56	**<0.001**
Style c4 × Melody c1	0.85	0.61 – 1.10	6.84	**<0.001**
Style c1 × Melody c2	0.16	-0.09 – 0.40	1.22	0.222
Style c2 × Melody c2	-0.02	-0.27 – 0.23	-0.15	0.879
Style c3 × Melody c2	-0.01	-0.26 – 0.23	-0.10	0.924
Style c4 × Melody c2	0.32	0.09 – 0.56	2.67	**0.008**
Style c1 × Melody c3	-0.43	-0.67 – -0.18	-3.40	**0.001**
Style c2 × Melody c3	0.26	0.00 – 0.51	1.98	**0.048**
Style c3 × Melody c3	1.09	0.81 – 1.37	7.74	**<0.001**
Style c4 × Melody c3	-0.93	-1.17 – -0.69	-7.74	**<0.001**
Style c1 × Melody c4	0.06	-0.18 – 0.31	0.50	0.620

Style c2 × Melody c4	-0.08	-0.32 – 0.17	-0.61	0.542
Style c3 × Melody c4	-0.47	-0.71 – -0.24	-3.89	**<0.001**
Style c4 × Melody c4	-0.08	-0.32 – 0.16	-0.67	0.502
Style c1 × Melody c5	-0.21	-0.45 – 0.03	-1.70	0.090
Style c2 × Melody c5	0.24	-0.01 – 0.49	1.92	0.055
Style c3 × Melody c5	0.66	0.41 – 0.91	5.11	**<0.001**
Style c4 × Melody c5	-0.02	-0.26 – 0.21	-0.19	0.848
Style c1 × Type [lyrics]	-0.05	-0.36 – 0.26	-0.33	0.744
Style c2 × Type [lyrics]	-0.03	-0.34 – 0.28	-0.17	0.862
Style c3 × Type [lyrics]	-0.07	-0.34 – 0.19	-0.55	0.579
Style c4 × Type [lyrics]	0.10	-0.16 – 0.36	0.77	0.441
Type [lyrics] × Melody c1	0.05	-0.20 – 0.29	0.37	0.713
Type [lyrics] × Melody c2	-0.04	-0.29 – 0.21	-0.32	0.748
Type [lyrics] × Melody c3	0.00	-0.25 – 0.25	0.02	0.988
Type [lyrics] × Melody c4	-0.01	-0.25 – 0.24	-0.05	0.962
Type [lyrics] × Melody c5	-0.00	-0.24 – 0.24	-0.01	0.992
ICC Stimulus	0.15			
ICC Participant	0.09			
ICC Singer	0.05			
N Participant	75			
N Stimulus	1316			
N Singer	22			
Observations	32900			
Marginal R^2 / Conditional R^2	0.093 / 0.355			

Note. Model syntax: lme4::glmer (Accuracy ~ 0 + Style + Type + Melody + Style:Melody + Style:Type + Type:Melody + (1 | Participant) + (1 | Stimulus) + (1 | Singer), family = binomial(link = “logit”))

Supplementary Figure S5

Accuracy of Style Recognition – All Main Effects and Interactions

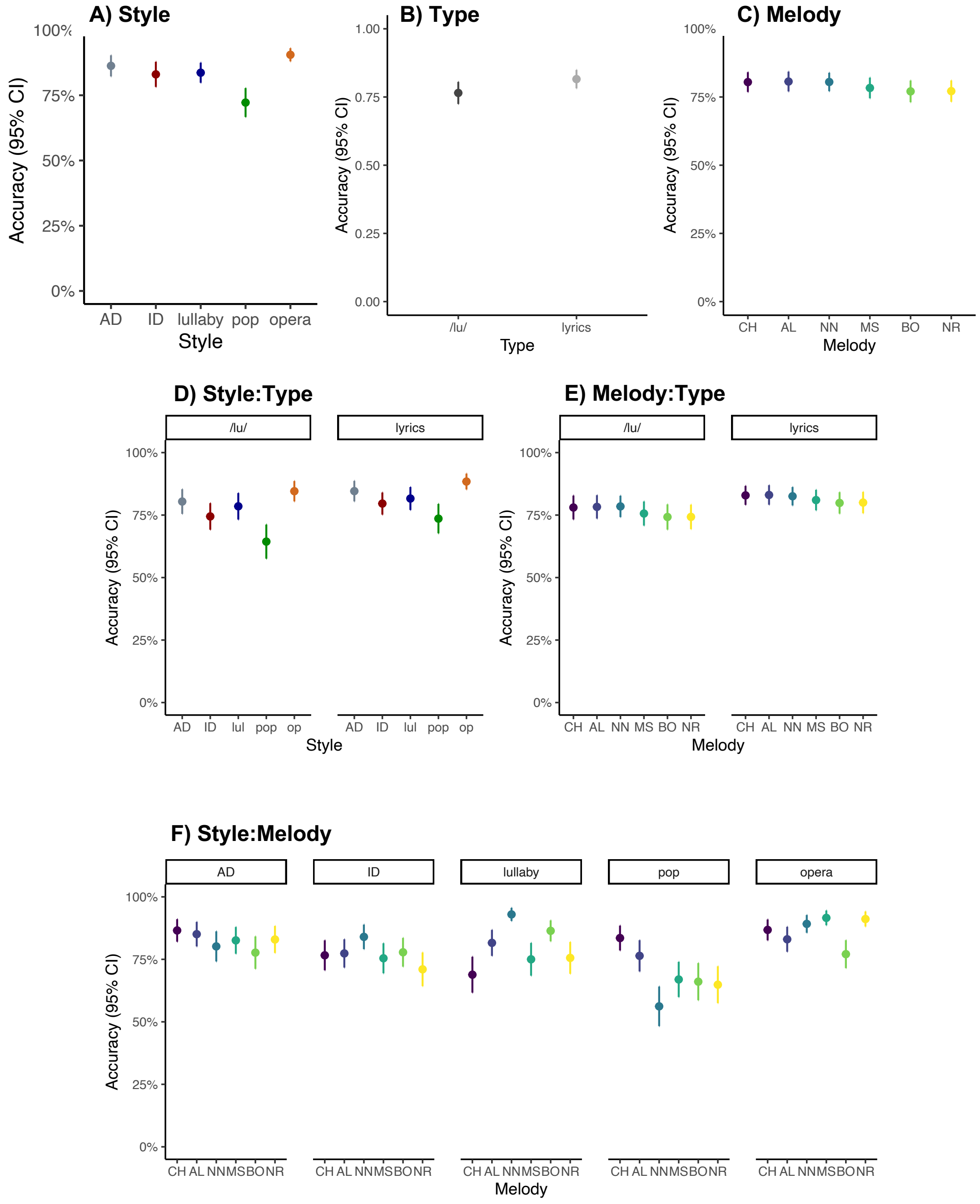

Note. Model-based predictions from the mixed logistic regression model described in Supplementary Table S2 (using the avg_predictions function from the marginaleffects R package). Error bars depict 95% confidence intervals. AD: adult-directed speech; ID: infant-directed speech; CH: Chove Chuva, AL: Alecrim Dourado; NN: Nana Nenê; MS: Melodia Sentimental; BO: Boi da Cara Preta; NR: Nesta Rua.

Supporting Text S2

Control experiment

Considering that we set different loudness levels for each singing style, we wondered how much the high style recognition found in the main experiment was related to the different levels of loudness. To investigate this, we conducted a control experiment in which all singing stimuli were normalized to the same loudness level.

Participants. Ten additional participants (6 self-reported as female, 3 as male, 1 undisclosed; M = 49.8 years old, SD = 19.2; 9 with German as mother tongue, 3 of which bilinguals, none of which with Portuguese as mother tongue) were recruited from the participant database of the Max Planck Institute for Empirical Aesthetics, in Frankfurt, Germany. They did not report having any hearing impairment and were lay listeners, with an average music sophistication score of 88.5 (SD = 10.6) according to the same 18-items adapted version of the scale of music sophistication of Gold-MSI (Müllensiefen et al., 2014). As before, participants provided written informed consent and were compensated at the rate of 14€ per hour of participation. The experimental procedure was ethically approved by the Ethics Council of the Max Planck Society, and participants were tested in the laboratories of the Max Planck Institute for Empirical Aesthetics, in Frankfurt, Germany.

Material. We used half of the singing performances of the main experiment, that is, a subset of 396 performances corresponding to the three melodies Nana Nenê, Chove Chuva, and Melodia Sentimental. Using the software To Audio Converter (version 1.0.16 – 1059), all stimuli were loudness normalized (following the EBU-R128 standard) to -23 LUFS.

Procedure. In relation to the main experiment, the only difference in procedure was that participants were presented both with performances with lyrics and with /lu/ - in different blocks of trials, and in counterbalanced order. As before, stimuli from different styles were presented intermixed and in random order. The task was the same forced-choice recognition task prosed to participants of Groups 1 and 2 of the main experiment, where participants had to indicate if singing performances sounded like a lullaby, a pop song, or an opera aria. We also included 20

repeated trials at the end of respective blocks (10 trials for stimuli with lyrics and 10 for stimuli with /lu/) so we could compute the test-retest intrarater agreement as before.

Statistical analyses. We repeated the analyses described for the main experiment: for each style and across all participants, we compared the proportion of accurate responses to chance-level performance (33% correct recognition) with Z-tests for proportions (one-tailed; aggregating performances with lyrics and with /lu/; and adjusting p-values to control the FWER with the Holm method). We compared results between experiments based both on the overall proportion of correct responses (CR) between both experiments (two-tailed Z-tests for proportions) and with pairwise comparisons between corresponding styles (two-tailed Z-tests for proportions, adjusting p-values to control the FWER of 3 comparisons with the Holm method). We calculated unbiased hit rates (Wagner, 1993), and conducted analysis of test-retest intrarater agreement based on repeated stimuli. We also fit the same mixed effects logistic model proposed for the main experiment, predicting accuracy at the individual trial level from the Style, the Type of performance and the Melody, and including random intercepts for participants, stimuli items and singers.

Results. The overall proportion of CR was 72.2%, which is lower than the 79% CR reported in the main experiment (χ^2 (1) = 94.8, $p < .001$). The proportion of CR was 81.6% for operatic, 70.7% for lullaby, and 64.5% for pop singing. These proportions are all above chance-level performance (adj. *p*s < .001). Unbiased hit rates were between 17.2% and 37.7% lower than proportions of CR: 67.5% for operatic, 51% for lullaby, and 40% for pop singing (unbiased chance level performance: 11% , 10.9%, and 11.5%, respectively).

Supplementary Figure S6 shows participants' style classification in the control experiment in relation to the presented stimuli. The majority of mistakes corresponded to participants answering that pop performances were lullabies, and vice-versa. Importantly, the proportion of CR by stimulus item was highly correlated between experiments ($r_{(394)} = .79$, $p < .001$), indicating that items that were well recognized in the main experiment also tended to be well recognized in the control experiment.

Supplementary Figure S6

Classification of Styles in the Control Experiment

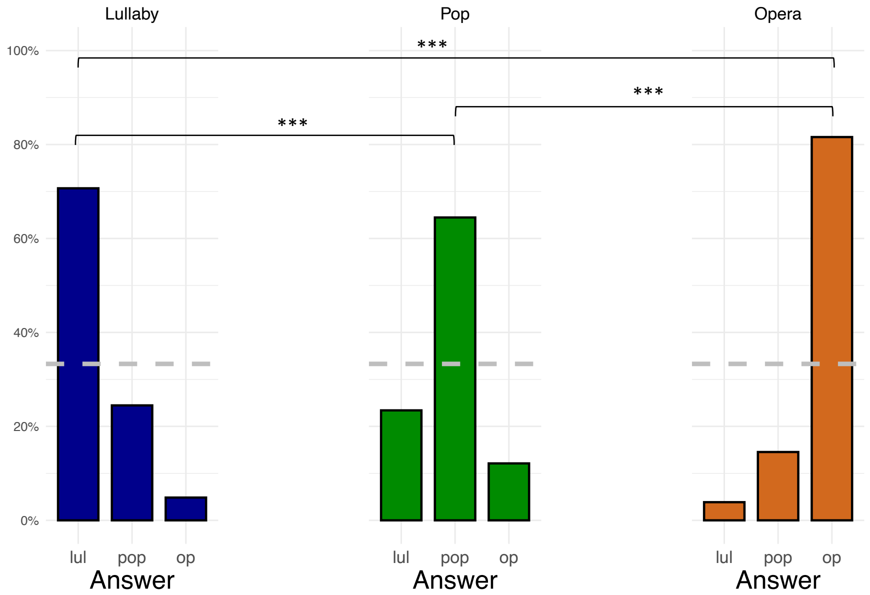

Note. In each plot, the y axis depicts the proportion of given responses in trials with different styles of vocalization. The dashed gray horizontal line represents chance-level performance (33%). lul: lullaby; op: opera.

We also fit the same mixed logistic model described for the main experiment to the data collected for this control experiment. The model revealed a significant effect of Style and interactions between Style and Melody and Style and Type. Please see Supplementary Table S4 for the model estimates and Supplementary Figure S7 for model-based predictions of all main effects and interactions.

Supplementary Table S4

Mixed Effects Logistic Regression of Accuracy of Style Recognition in the Control Experiment

Predictors	*Log-Odds*	*CI*	*Statistic*	*p*
Style c [lullaby]	1.35	0.80 – 1.90	4.78	**<0.001**
Style c [opera]	1.75	1.20 – 2.31	6.17	**<0.001**
Style c [pop]	0.58	0.04 – 1.12	2.09	**0.037**
Type [lyrics]	0.04	-0.14 – 0.22	0.47	0.640
Melody c1	-0.03	-0.21 – 0.15	-0.36	0.716
Melody c2	-0.11	-0.29 – 0.07	-1.23	0.219
Style c1 × Melody c1	-0.62	-0.80 – -0.44	-6.74	**<0.001**
Style c2 × Melody c1	-0.23	-0.41 – -0.05	-2.44	**0.015**
Style c1 × Melody c2	-0.71	-0.89 – -0.53	-7.67	**<0.001**
Style c2 × Melody c2	0.45	0.26 – 0.64	4.57	**<0.001**
Style c1 × Type [lyrics]	-0.31	-0.57 – -0.06	-2.38	**0.017**
Style c2 × Type [lyrics]	-0.01	-0.27 – 0.26	-0.04	0.968
Type [lyrics] × Melody c1	-0.01	-0.26 – 0.25	-0.06	0.953
Type [lyrics] × Melody c2	0.10	-0.16 – 0.35	0.75	0.451
ICC Stimulus	0.041			
ICC Participant	0.142			
ICC Singer	0.031			
N Participant	10			
N Stimulus	396			
N Singer	22			
Observations	3960			
Marginal R^2 / Conditional R^2	0.151 / 0.333			

Note. Model syntax: lme4::glmer (Accuracy ~ 0 + Style + Type + Melody + Style:Melody + Style:Type + Type:Melody + (1 | Participant) + (1 | Stimulus) + (1 | Singer), data, family = binomial(link = "logit"))

Supplementary Figure S7

Accuracy of Style Recognition in the Control Experiment

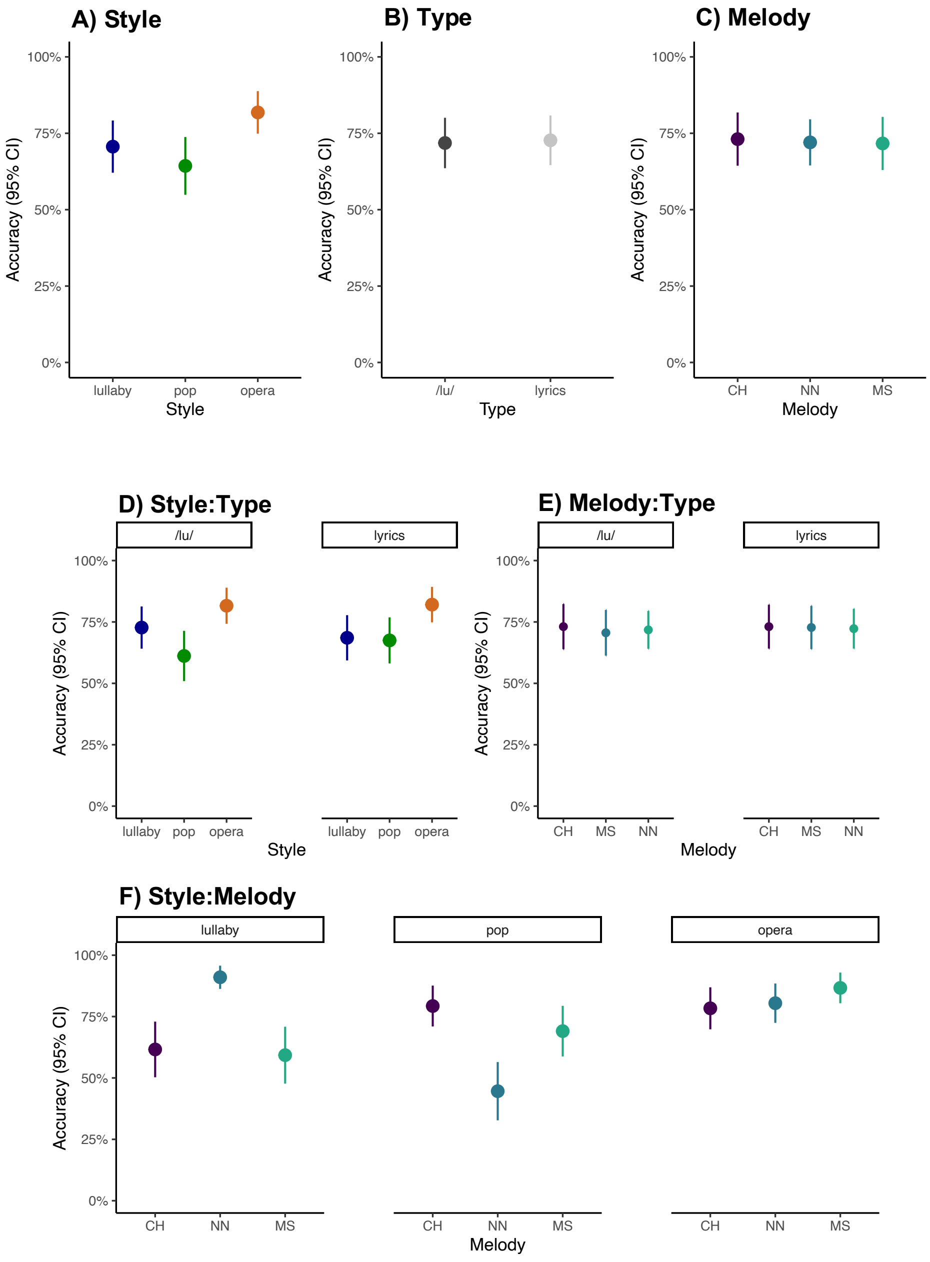

Note. Model-based predictions from the mixed logistic regression model described in Supplementary Table S4 (using the avg_predictions function from the marginal effects R package). Error bars depict 95% confidence intervals. AD: adult-directed speech; ID: infant-directed speech; CH: Chove Chuva, NN: Nana Nenê; MS: Melodia Sentimental.

Interestingly, the interaction between Style and Melody replicated the observation made in the main experiment that melodies were better recognized when performed in a style congruent to their original genre: as portrayed in Supplementary Figure S7F, the melody Nana Nenê, originally a lullaby, was better recognized when performed as a lullaby; Chove Chuva, originally a pop "MPB" song, was better recognized when performed as a pop song; and Melodia Sentimental, originally an art song, was better recognized when performed as an opera aria.

The proportion of correct recognition by each participant ranged from 43.9% to 88.4% (see Supplementary Figure 8), confirming that recognition was above chance level for all participants, and that most participants could do the task with good accuracy.

Supplementary Figure S8

Proportion of Correct Recognition by Participant in the Control Experiment

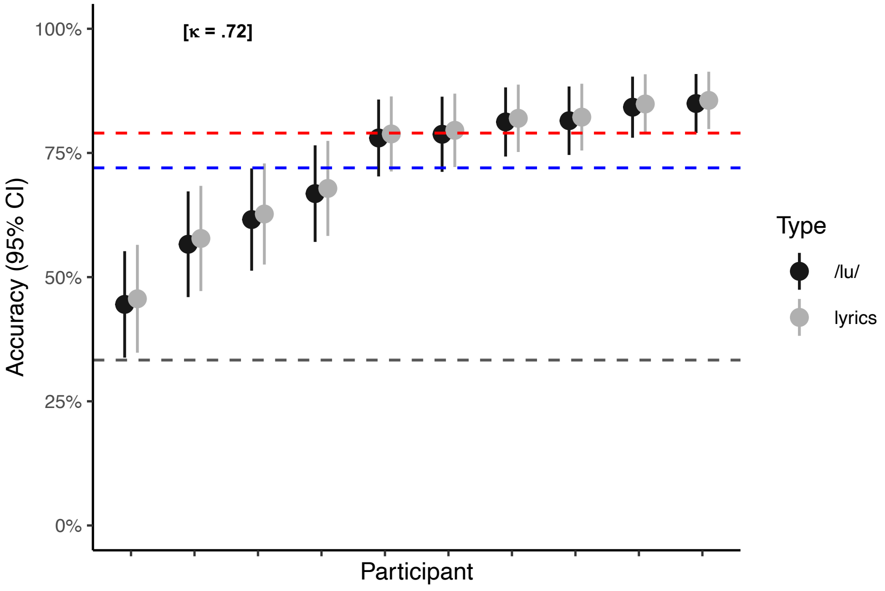

Note. N = 10 participants. Model-based predictions from the mixed logistic regression model described in Supplementary Table S4 (using the avg_predictions function from the marginal effects R package). Error bars depict 95% confidence intervals. The gray dashed horizontal line (bottom line) represents chance-level performance. The red dashed horizontal line (top line) represents the average proportion of correct recognition in the main experiment, and the blue dashed horizontal line (middle line) represents the average proportion of correct recognition in

the control experiment. The value between brackets represents intrarater test-retest agreement (at the group level) as measured by Cohens' kappa.

In summary, the slightly higher rate of correct style recognition in the main experiment suggests that the different loudness levels between styles in that particular experiment aided participants' style recognition. Nevertheless, the (still) high style recognition rate in the control experiment indicates that the difference in loudness levels was not a critical factor for accurate style recognition, that is, that other perceptual features were enough to guide participants in their style recognition. We refer interested readers to Bruder and Larrouy-Maestri (2023) for a more detailed comparison of the main and the control experiments, as well as exploratory analyses on the role of acoustic features in the perceptual categorization of different singing styles.

Supporting Text S3

Extraction of additional acoustic descriptors

Soundgen. A total of 163 features were extracted, presented as mean, median, and standard deviation summaries per file. We used the analyze function for batch extraction (soundgen::analyze(wav_list, pitchFloor = 85, pitchCeiling = 800, samplingRate = 44100, windowLength = 20). Please refer to Anikin (2019) for further details about Soundgen, and to the developers' website (http://cogsci.se/soundgen/acoustic_analysis.html) for a summary of these features. Here is a list with the names of extracted features: duration, duration_noSilence, voiced, voiced_noSilence, amEnvDep_mean, amEnvDep_median, amEnvDep_sd, amEnvDepVoiced_mean, amEnvDepVoiced_median, amEnvDepVoiced_sd, amEnvFreq_mean, amEnvFreq_median, amEnvFreq_sd, amEnvFreqVoiced_mean, amEnvFreqVoiced_median, amEnvFreqVoiced_sd, amMsFreq_mean, amMsFreq_median, amMsFreq_sd, amMsFreqVoiced_mean, amMsFreqVoiced_median, amMsFreqVoiced_sd, amMsPurity_mean, amMsPurity_median, amMsPurity_sd, amMsPurityVoiced_mean, amMsPurityVoiced_median, amMsPurityVoiced_sd, ampl_mean, ampl_median, ampl_sd, ampl_noSilence_mean, ampl_noSilence_median, ampl_noSilence_sd, amplVoiced_mean, amplVoiced_median, amplVoiced_sd, CPP_mean, CPP_median, CPP_sd, dom_mean, dom_median, dom_sd, domVoiced_mean, domVoiced_median, domVoiced_sd, entropy_mean, entropy_median, entropy_sd, entropySh_mean, entropySh_median, entropySh_sd, entropyShVoiced_mean, entropyShVoiced_median, entropyShVoiced_sd, entropyVoiced_mean, entropyVoiced_median, entropyVoiced_sd, f1_freq_mean, f1_freq_median, f1_freq_sd, f1_width_mean, f1_width_median, f1_width_sd, f2_freq_mean, f2_freq_median, f2_freq_sd, f2_width_mean, f2_width_median, f2_width_sd, f3_freq_mean, f3_freq_median, f3_freq_sd, f3_width_mean, f3_width_median, f3_width_sd, flux_mean, flux_median, flux_sd, fmDep_mean, fmDep_median, fmDep_sd, fmFreq_mean, fmFreq_median, fmFreq_sd, fmPurity_mean, fmPurity_median, fmPurity_sd, harmEnergy_mean, harmEnergy_median, harmEnergy_sd, harmHeight_mean, harmHeight_median, harmHeight_sd, HNR_mean, HNR_median, HNR_sd, HNRVoiced_mean, HNRVoiced_median, HNRVoiced_sd, loudness_mean, loudness_median, loudness_sd, loudnessVoiced_mean, loudnessVoiced_median, loudnessVoiced_sd, novelty_mean, novelty_median, novelty_sd, noveltyVoiced_mean, noveltyVoiced_median,

noveltyVoiced_sd, peakFreq_mean, peakFreq_median, peakFreq_sd, peakFreqVoiced_mean, peakFreqVoiced_median, peakFreqVoiced_sd, pitch_mean, pitch_median, pitch_sd, quartile25_mean, quartile25_median, quartile25_sd, quartile25Voiced_mean, quartile25Voiced_median, quartile25Voiced_sd, quartile50_mean, quartile50_median, quartile50_sd, quartile50Voiced_mean, quartile50Voiced_median, quartile50Voiced_sd, quartile75_mean, quartile75_median, quartile75_sd, quartile75Voiced_mean, quartile75Voiced_median, quartile75Voiced_sd, roughness_mean, roughness_median, roughness_sd, roughnessVoiced_mean, roughnessVoiced_median, roughnessVoiced_sd, specCentroid_mean, specCentroid_median, specCentroid_sd, specCentroidVoiced_mean, specCentroidVoiced_median, specCentroidVoiced_sd, specSlope_mean, specSlope_median, specSlope_sd, specSlopeVoiced_mean, specSlopeVoiced_median, specSlopeVoiced_sd, subDep_mean, subDep_median, subDep_sd, subRatio_mean, subRatio_median, subRatio_sd

Essentia. While exploring possibly useful features to characterize our stimuli, we also extracted multiple features using the Essentia toolbox (Bogdanov et al., 2013), an open-source C++ library for music information retrieval (MIR). While we do not use them in our characterization of the vocalizations, we also make them available with the hope that they may be useful to other researchers (e.g., when comparing toolboxes for audio signal description). We used the out-of-box executable streaming extractor freesound (https://essentia.upf.edu/freesound_extractor.html) to extract the following low-level features: average_loudness, barkbands_kurtosis, barkbands_skewness, barkbands_spread, dissonance, hfc, pitch, pitch_instantaneous_confidence, pitch_salience, silence_rate_20dB, silence_rate_30dB, silence_rate_60dB, spectral_complexity, spectral_crest, spectral_decrease, spectral_energy, spectral_energyband_high, spectral_energyband_low, spectral_energyband_middle_high, spectral_energyband_middle_low, spectral_entropy, spectral_flatness_db, spectral_flux, spectral_rms, spectral_rolloff, spectral_skewness, spectral_spread, spectral_centroid, spectral_kurtosis, spectral_strongpeak, zerocrossingrate, barkbands (01-27), frequency_bands (01-27), gfcc (01-13), mfcc (01-13), scvalleys (01-06), spectral_contrast (01-06). Note that barkbands and frequency_bands refer to 27 spectral subbands, gfcc and mfcc refer to 13 spectral subbands, and scvalleys and spectral_contrast to six subbands. For each feature, an average value and standard deviation were extracted, and in some cases also the variance of the derivative of a feature (dvar). We excluded all barkbands.dmean

measures, which were perfectly correlated with frequency_bands.dmean measures, and report a total of 186 features. Please refer to https://essentia.upf.edu/freesound_extractor.html for a description of algorithms.

VoiceSauce. In addition to the measures reported in the main text, we also report VoiceSauce's Energy measure (Root Mean Square Energy), generally used to evaluate the amplitude of the audio signal; and various formant estimations (pF1_mean, pF2_mean, pF3_mean, pF4_mean) based on Praat's Burg algorithm (and using the same settings of pitch_floor = 85 and pitch_ceiling = 600 Hz as in other analyses). Please see Shue et al. (2011) for more information about VoiceSauce.

Supplementary Figure S9

Screeplot of Principal Component Analysis

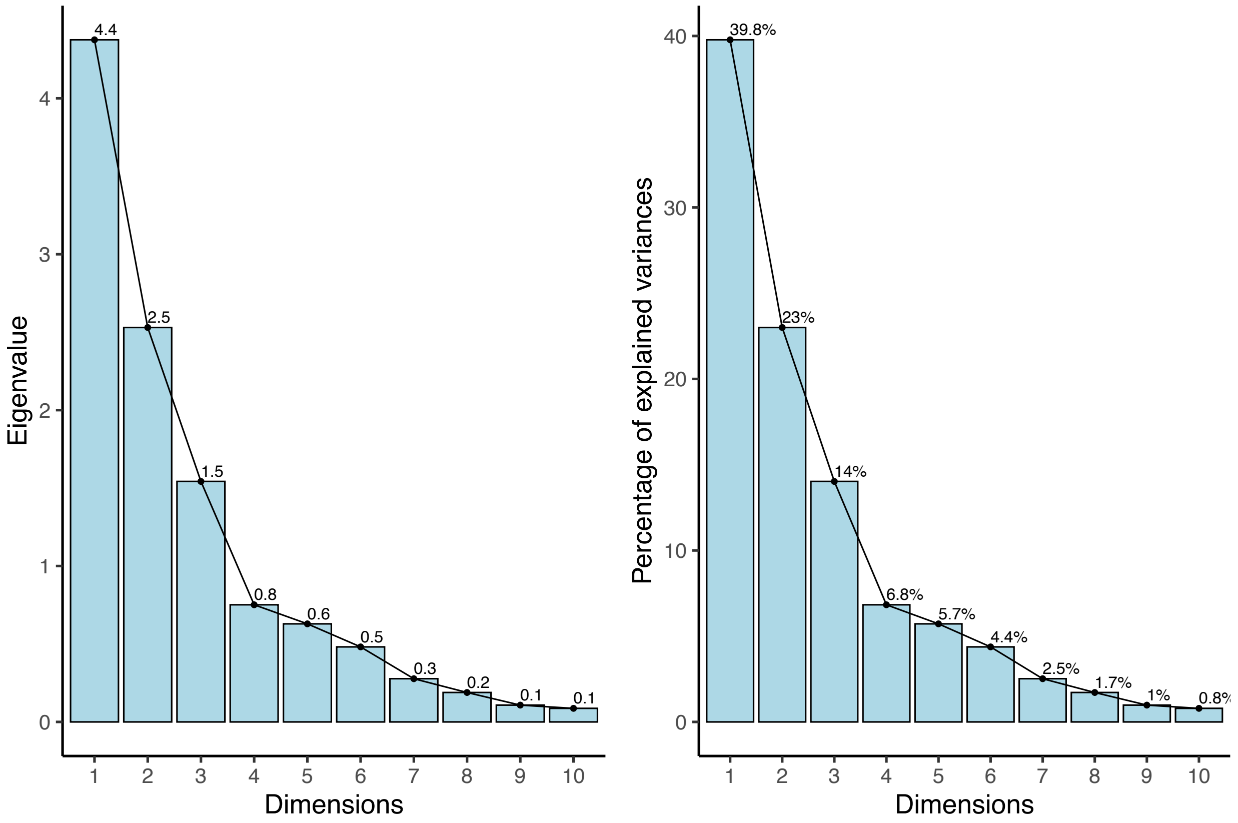

Note. Top: Scree plot illustrating the eigenvalues for each component. The first two dimensions combined explain 62.8% of the variance; the first three dimensions combined explain 76.8% of the variance.

Supplementary Table S5

Variable Contribution to the Principal Component Analysis

Variable	PC1	PC2	PC3
Jitter (local)	**19.7**	0.7	0.5
HNR35	**19.1**	2.2	0.0
Shimmer (local)	**17.7**	3.2	0.1
Syllable rate	**11.0**	0.1	0.1
amEnvDep	**10.4**	8.5	0.4
Mean f_o	7.7	**13.6**	**11.5**
harmHeight	6.1	**25.3**	2.0
harmEnergy	4.5	5.5	**24.0**
fmDep	2.4	**16.0**	2.9
CPP	1.4	**14.1**	**31.0**
SD f_o	0.1	**10.9**	**27.4**

Note. Contribution of each acoustic feature to the first, second and third dimensions of the PCA. The threshold for significant contribution is set at 9.1%, represented as the expected value if all 11 variables contributed equally to that dimension. Variables contributing above this threshold are highlighted in bold. amDep: depth of amplitude modulation; HNR35: harmonics-to-noise ratio between 0 and 3.5kHz; harmEnergy: energy in harmonics; fmDep: depth of frequency modulation; harmHeight: Height in harmonics; SD f_o: standard deviation of the fundamental frequency; Mean f_o: average fundamental frequency; CPP: cepstral peak prominence.

Supplementary Figure S10

Distribution of Acoustic Features by Melody

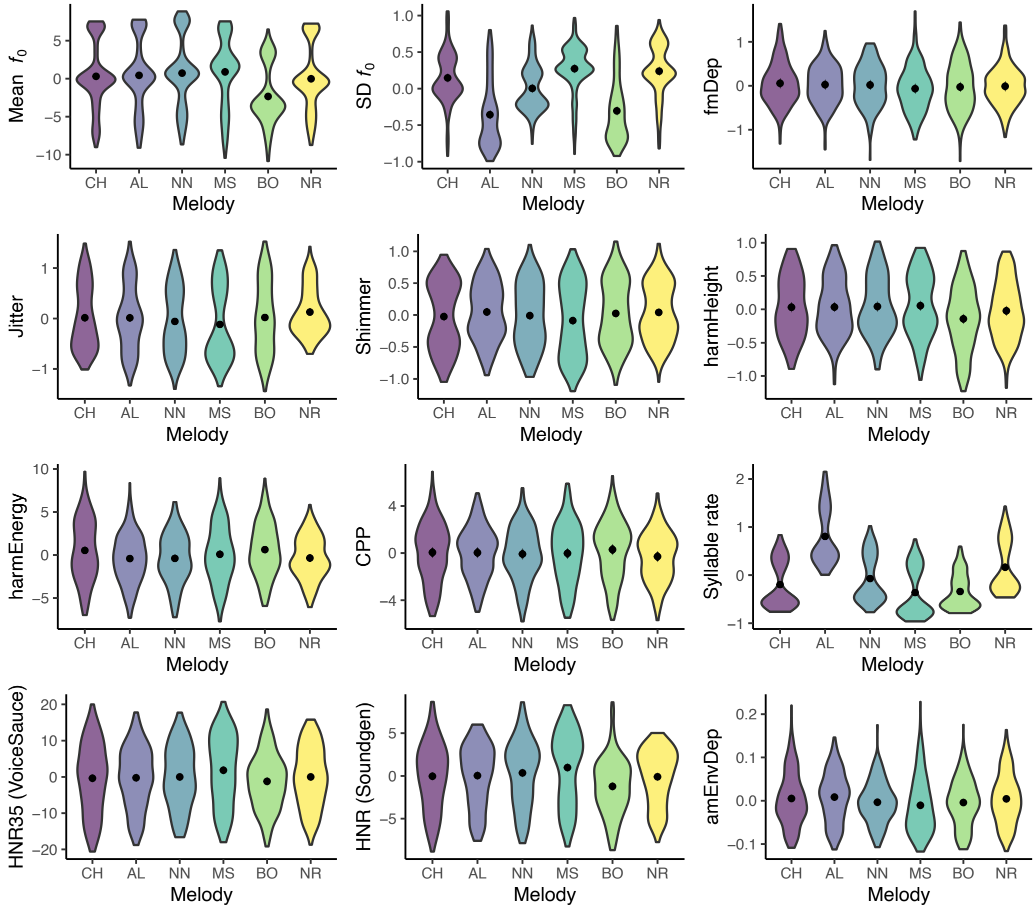

Note. N = 1320 performances (220 per melody). Violin plots show the distribution of (zero-centered) acoustic features by melody. Dots and error bars in the center of each distribution represent model-based estimates and 95% confidence intervals (using the marginaleffects::predictions function). CH: Chove Chuva, AL: Alecrim Dourado; NN: Nana Nenê; MS: Melodia Sentimental; BO: Boi da Cara Preta; NR: Nesta Rua. f_o: fundamental frequency; SD: standard deviation; fmDep: depth of frequency modulation; harmHeight: harmonic height; harmEnergy: harmonic energy; CPP: cepstral peak prominence; HNR35: harmonics-to-noise ratio between 0-3.5kHz (VoiceSauce); HNR: harmonics-to-noise ratio (Soundgen); amEnvDep: depth of amplitude modulation; fmDep: depth of frequency modulation. Please see Supplementary Table S6 for a summary of untransformed values of each acoustic feature.

Supplementary Table S6

Summary Descriptive Statistics of Acoustic Features by Style of Vocalization, by Type of Production and by Melody

Style	AD		ID		lullaby		pop		opera	
	Mean	*SD*	Mean	*SD*	Mean	*SD*	Mean	*SD*	Mean	*SD*
f_o mean (Hz)	247	24.9	350	67.1	342	31	341	31.1	502	38.4
f_o SD (Hz)	48.9	17.4	85.6	26	50.2	19	50.6	18.7	73.9	25.6
Jitter (local)	0.013	0.005	0.014	0.005	0.005	0.002	0.005	0.002	0.005	0.002
Shimmer (local)	0.071	0.018	0.069	0.017	0.026	0.007	0.03	0.011	0.042	0.013
CPP (dB)	20.9	1.37	19.8	1.98	17.9	1.5	22.5	1.83	21.8	1.45
HNR35-VS	34.4	5.69	36.3	5.94	42	5.85	48	5.38	51.9	4.75
HNR-Soundgen	15.4	1.97	17.6	2.41	22.3	2.33	21.6	2.65	22.8	1.95
Syllable rate (syl/sec)	2.1	0.5	2.2	0.5	1.2	0.4	1.3	0.4	1.2	0.4
harmEnergy (dB)	2.26	2.46	0.253	2.72	-2.19	2.57	1.3	3.24	0.038	2.17
harmHeight (Hz)	3921	1012	5024	1747	3619	947	6825	1854	9318	1997
amDep (0-1)	0.20	0.05	0.22	0.05	0.13	0.04	0.13	0.04	0.18	0.03
fmDep (semitones)	0.31	0.15	0.39	0.19	0.19	0.06	0.26	0.09	0.40	0.18

Type of production	/lu/		lyrics	
	Mean	*SD*	Mean	*SD*
f_o mean (Hz)	358	91.6	354	92.4
f_o SD (Hz)	60.6	26	63.1	26.7
Jitter (local)	0.006	0.004	0.01	0.006
Shimmer (local)	0.043	0.021	0.052	0.025
CPP (dB)	20.5	2.46	20.6	2.17
HNR35-VS	46.1	7.3	39	8.5
HNR-Soundgen	21.2	3.46	18.7	3.53
Syllable rate (syl/sec)	1.6	0.6	1.6	0.6
harmEnergy (dB)	-1.65	2.19	2.32	2.42
harmHeight (Hz)	5642	2636	5841	2629
amDep (0-1)	0.15	0.05	0.2	0.05
fmDep (semitones)	0.3	0.17	0.32	0.16

Melody	CH		AL		NN		MS		BO		NR	
	**Mean**	***SD***	**Mean**	***SD***	**Mean**	***SD***	**Mean**	***SD***	**Mean**	***SD***	**Mean**	***SD***
**f_o mean (Hz)**	362.0	90.1	365.0	94.5	372.0	98.7	375.0	90.3	309.0	71.3	355.0	89.3
**f_o SD (Hz)**	69.1	24.6	44	23.4	59.3	19.8	77.4	21.5	45.9	23.4	75.5	24
**Jitter (local)**	0.01	0.01	0.01	0.01	0.01	0.01	0.01	0.01	0.01	0.01	0.01	0.01
**Shimmer (local)**	0.05	0.02	0.05	0.02	0.05	0.02	0.05	0.03	0.05	0.03	0.05	0.02
**CPP (dB)**	20.6	2.45	20.6	2.12	20.5	2.22	20.6	2.54	20.9	2.45	20.3	2.08
**HNR35-VS**	42.2	9.54	42.3	8.31	42.5	8.47	44.4	9.62	41.3	7.56	42.5	8.16
**HNR-Soundgen**	19.9	3.82	20	3.6	20.3	3.79	20.9	4.22	18.7	3.28	19.9	3.2
**Syllable rate (syl/sec)**	1.4	0.5	2.4	0.6	1.5	0.5	1.2	0.5	1.2	0.4	1.7	0.5
**harmEnergy (dB)**	0.85	3.5	-0.1	2.83	-0.1	2.84	0.4	3.31	0.94	3.05	-0	2.46
**harmHeight (Hz)**	5910	2674	5877	2582	5974	2761	6087	2789	5028	2375	5573	2479
**amDep (0-1)**	0.18	0.06	0.18	0.06	0.17	0.05	0.16	0.07	0.17	0.06	0.18	0.06
**fmDep (semitones)**	0.33	0.18	0.31	0.14	0.31	0.15	0.29	0.18	0.31	0.18	0.3	0.15

Note. f_o: fundamental frequency; *SD*: standard deviation; CPP: Cepstral peak prominence; HNR35-VS: harmonics-to-noise ratio (0 - 3.5 kHz; from VoiceSauce); HNR-Soundgen: harmonics-to-noise ratio (from Soundgen); fmDep: depth of frequency modulation; harmHeight: harmonic height; harmEnergy: harmonic energy; amDep: depth of amplitude modulation; fmDep: depth of frequency modulation. CH: Chove Chuva, AL: Alecrim Dourado; NN: Nana Nenê; MS: Melodia Sentimental; BO: Boi da Cara Preta; NR: Nesta Rua.

Supplementary Figure S11

Correlation matrix of acoustic features

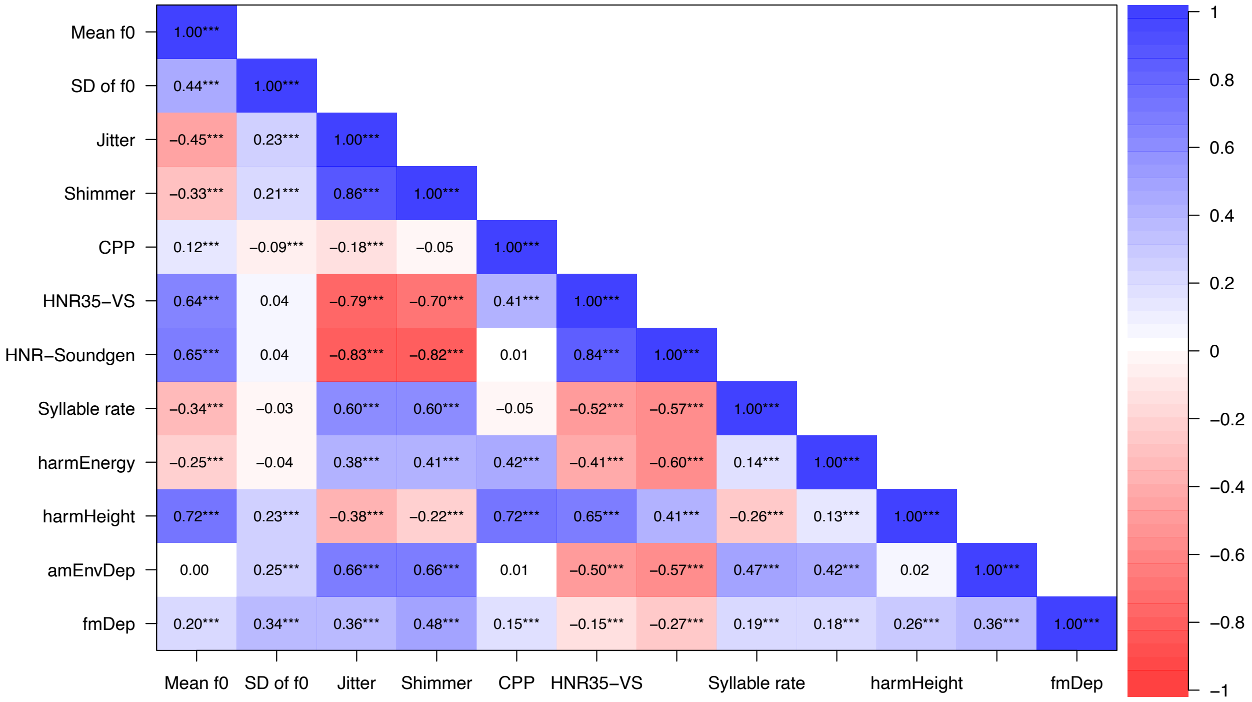

Note. f_o: fundamental frequency; *SD*: standard deviation; CPP: Cepstral peak prominence; HNR35-VS: harmonics-to-noise ratio (0 - 3.5 kHz; from VoiceSauce); HNR-Soundgen: harmonics-to-noise ratio (from Soundgen); fmDep: depth of frequency modulation; harmHeight: harmonic height; harmEnergy: harmonic energy; amDep: depth of amplitude modulation; fmDep: depth of frequency modulation.

Supplementary Table S7

Pairwise Comparisons for Effect of Style for each Acoustic Feature

Style 1	Style 2	Estimate	Std.error	Statistic	p.adj
		**f_0 mean**			
ID	AD	5.810611	0.601752	9.656152	<0.001
lullaby	AD	5.657002	0.292127	19.3649	<0.001
lullaby	ID	-0.15361	0.607716	-0.25276	0.800
opera	AD	12.34184	0.290805	42.44024	<0.001
opera	ID	6.531231	0.594765	10.9812	<0.001
opera	lullaby	6.68484	0.079243	84.35925	<0.001
opera	pop	6.723434	0.076148	88.2945	<0.001
pop	AD	5.618408	0.290823	19.31898	<0.001
pop	ID	-0.1922	0.603593	-0.31843	0.750
pop	lullaby	-0.03859	0.075559	-0.51078	0.610
		**f_0 SD**			
ID	AD	0.565674	0.053586	10.55634	<0.001
lullaby	AD	0.001917	0.053918	0.035555	0.972
lullaby	ID	-0.56376	0.055299	-10.1947	<0.001
opera	AD	0.401247	0.054488	7.363938	<0.001
opera	ID	-0.16443	0.056363	-2.91731	0.004
opera	lullaby	0.39933	0.01229	32.49295	<0.001
opera	pop	0.387545	0.012584	30.79591	<0.001
pop	AD	0.013701	0.051705	0.264993	0.791
pop	ID	-0.55197	0.0554	-9.96341	<0.001
pop	lullaby	0.011784	0.012537	0.93994	0.347
		**Jitter (local)**			
ID	AD	0.025386	0.060033	0.422869	0.672
lullaby	AD	-1.06468	0.053909	-19.7497	<0.001
lullaby	ID	-1.09006	0.061767	-17.6479	<0.001
opera	AD	-1.06951	0.059739	-17.9029	<0.001
opera	ID	-1.09489	0.062948	-17.3935	<0.001
opera	lullaby	-0.00483	0.048382	-0.0998	0.921
opera	pop	0.004816	0.040803	0.118041	0.906
pop	AD	-1.07432	0.053506	-20.0786	<0.001
pop	ID	-1.09971	0.060104	-18.2967	<0.001
pop	lullaby	-0.00964	0.041006	-0.23521	0.814

		**Shimmer (local)**			
ID	AD	-0.02321	0.043052	-0.53911	0.590
lullaby	AD	-1.00341	0.053496	-18.7565	<0.001
lullaby	ID	-0.9802	0.041324	-23.7197	<0.001
opera	AD	-0.54796	0.065832	-8.3236	<0.001
opera	ID	-0.52475	0.067497	-7.77446	<0.001
opera	lullaby	0.455443	0.068203	6.677743	<0.001
opera	pop	0.312159	0.058079	5.3747	<0.001
pop	AD	-0.86012	0.048851	-17.6069	<0.001
pop	ID	-0.83691	0.043412	-19.2785	<0.001
pop	lullaby	0.143284	0.033894	4.227447	<0.001
		**fmDep**			
ID	AD	0.228952	0.059626	3.839819	<0.001
lullaby	AD	-0.40612	0.077422	-5.2455	<0.001
lullaby	ID	-0.63507	0.060791	-10.4468	<0.001
opera	AD	0.265298	0.082388	3.220101	0.001
opera	ID	0.036346	0.05508	0.659883	0.509
opera	lullaby	0.671418	0.069203	9.70213	<0.001
opera	pop	0.390256	0.06249	6.245051	<0.001
pop	AD	-0.12496	0.061859	-2.02006	0.043
pop	ID	-0.35391	0.055516	-6.37488	<0.001
pop	lullaby	0.281162	0.042885	6.556196	<0.001
		**Height of harmonics**			
ID	AD	0.222783	0.065197	3.417053	<0.001
lullaby	AD	-0.08077	0.048117	-1.6785	0.093
lullaby	ID	-0.30355	0.072354	-4.19531	<0.001
opera	AD	0.876151	0.049639	17.65061	<0.001
opera	ID	0.653368	0.060808	10.7447	<0.001
opera	lullaby	0.956916	0.055368	17.28288	<0.001
opera	pop	0.32434	0.044153	7.345779	<0.001
pop	AD	0.551811	0.051171	10.78358	<0.001
pop	ID	0.329028	0.067149	4.899964	<0.001
pop	lullaby	0.632576	0.044936	14.07735	<0.001
		**Cepstral Peak Prominence**			
ID	AD	-1.12852	0.413946	-2.72624	0.006
lullaby	AD	-3.04947	0.233897	-13.0377	<0.001
lullaby	ID	-1.92095	0.410941	-4.67452	<0.001
opera	AD	0.877424	0.278655	3.148778	0.002
opera	ID	2.005939	0.434227	4.619563	<0.001
opera	lullaby	3.92689	0.341744	11.49073	<0.001
opera	pop	-0.74224	0.31354	-2.3673	0.018
pop	AD	1.619667	0.283194	5.719287	<0.001
pop	ID	2.748182	0.443447	6.197311	<0.001
pop	lullaby	4.669133	0.300248	15.55093	<0.001

HNR35-VS

ID	AD	1.895379	0.788882	2.402614	0.016
lullaby	AD	7.5865	0.980908	7.734157	<0.001
lullaby	ID	5.691121	0.986566	5.768619	<0.001
opera	AD	17.49521	0.818002	21.38773	<0.001
opera	ID	15.59983	0.942007	16.5602	<0.001
opera	lullaby	9.908712	0.966565	10.25147	<0.001
opera	pop	3.857295	0.697289	5.531849	<0.001
pop	AD	13.63792	0.703852	19.3761	<0.001
pop	ID	11.74254	0.752134	15.6123	<0.001
pop	lullaby	6.051417	0.670277	9.02823	<0.001

HNR- Soundgen

ID	AD	2.222059	0.367871	6.040324	<0.001
lullaby	AD	6.96858	0.369015	18.88428	<0.001
lullaby	ID	4.746521	0.352663	13.45909	<0.001
opera	AD	7.447891	0.333636	22.32341	<0.001
opera	ID	5.225833	0.429902	12.15586	<0.001
opera	lullaby	0.479311	0.338255	1.417012	0.156
opera	pop	1.191989	0.274665	4.339793	<0.001
pop	AD	6.255902	0.26841	23.30724	<0.001
pop	ID	4.033844	0.308736	13.06568	<0.001
pop	lullaby	-0.71268	0.180394	-3.95067	<0.001

Syllable rate

ID	AD	0.105456	0.039924	2.641413	0.008
lullaby	AD	-0.87925	0.042031	-20.9192	<0.001
lullaby	ID	-0.9847	0.048752	-20.1983	<0.001
opera	AD	-0.91471	0.040968	-22.3276	<0.001
opera	ID	-1.02017	0.049461	-20.6259	<0.001
opera	lullaby	-0.03547	0.015052	-2.35626	0.018
opera	pop	-0.11313	0.011814	-9.57621	<0.001
pop	AD	-0.80158	0.040941	-19.5788	<0.001
pop	ID	-0.90704	0.049051	-18.4916	<0.001
pop	lullaby	0.077667	0.012042	6.449554	<0.001

harmEnergy

ID	AD	-2.01188	0.437015	-4.6037	<0.001
lullaby	AD	-4.45575	0.383861	-11.6077	<0.001
lullaby	ID	-2.44386	0.21569	-11.3305	<0.001
opera	AD	-2.22677	0.37855	-5.88238	<0.001
opera	ID	-0.21489	0.470395	-0.45682	0.648
opera	lullaby	2.228974	0.388824	5.732602	<0.001
opera	pop	-1.26104	0.292649	-4.30907	<0.001
pop	AD	-0.96573	0.276201	-3.49646	<0.001
pop	ID	1.046158	0.357961	2.922548	0.003
pop	lullaby	3.490019	0.286295	12.19027	<0.001

	amDep				
ID	AD	0.022117	0.006451	3.428378	<0.001
lullaby	AD	-0.07092	0.004991	-14.2103	<0.001
lullaby	ID	-0.09304	0.005961	-15.6086	<0.001
opera	AD	-0.01605	0.005629	-2.85207	0.004
opera	ID	-0.03817	0.005569	-6.85461	<0.001
opera	lullaby	0.05487	0.003337	16.44133	<0.001
opera	pop	0.053432	0.00309	17.29401	<0.001
pop	AD	-0.06949	0.004441	-15.6451	<0.001
pop	ID	-0.0916	0.005382	-17.0199	<0.001
pop	lullaby	0.001438	0.002577	0.557828	0.577

Note. Pairwise comparisons between Styles obtained with the avg_comparisons function from the marginaleffects R package, based on linear mixed models predicting each acoustic feature from Style, Type of production, Melody, and their two-way interactions (acoustic feature ~ 0 + Style:Melody + Style:Type + Melody:Type + (1 + Style | Singer)). p.adj: p-values adjusted with the Holm method; AD: adult-directed; ID: infant-directed; f_o: fundamental frequency; SD: standard deviation. CPP: Cepstral peak prominence; HNR35-VS: harmonics-to-noise ratio (0 - 3.5 kHz; from VoiceSauce); HNR-Soundgen: harmonics-to-noise ratio (from Soundgen); fmDep: depth of frequency modulation; harmHeight: harmonic height; harmEnergy: harmonic energy; amDep: depth of amplitude modulation; fmDep: depth of frequency modulation.

References

Anikin, A. (2019). Soundgen: An open-source tool for synthesizing nonverbal vocalizations. *Behavior Research Methods*, *51*(2), 778–792. https://doi.org/10.3758/s13428-018-1095-7

Bogdanov, D., Wack, N., Gomez, E., Gulati, S., Herrera, P., & Serra, X. (2013). Essentia: An audio analysis library for music information retrieval. *Proceedings of the 14th International Society for Music Information Retrieval Conference*, 493–498. https://ismir2013.ismir.net/wp-content/uploads/2013/10/Proceedings-ISMIR2013-Final.pdf

Bruder, C., & Larrouy-Maestri, P. (2023). Classical singers are also proficient in non-classical singing. *Frontiers in Psychology*, *14*. https://doi.org/10.3389/fpsyg.2023.1215370

Müllensiefen, D., Gingras, B., Musil, J., & Stewart, L. (2014). The musicality of non-musicians: An index for assessing musical sophistication in the general population. *PLoS ONE*, *9*(2), e89642. https://doi.org/10.1371/journal.pone.0089642

Shue, Y. L., Keating, P., Vicenik, C., & Yu, K. (2011). VoiceSauce: A program for voice analysis. *Proceedings of the ICPhS XVII, 1846-1849*. https://linguistics.ucla.edu/people/keating/Shue-etal_ICPhS_2011.pdf

Wagner, H. L. (1993). On measuring performance in category judgment studies of nonverbal behavior. *Journal of Nonverbal Behavior*, *17*(1), 3–28. https://doi.org/10.1007/BF00987006
